# What It Takes for Imidazolium Cations to Promote Electrochemical Reduction of CO_2_

**DOI:** 10.1021/acsenergylett.2c01372

**Published:** 2022-09-15

**Authors:** Sobhan Neyrizi, Joep Kiewiet, Mark A. Hempenius, Guido Mul

**Affiliations:** † Photocatalytic Synthesis Group, Faculty of Science & Technology, University of Twente, P.O. Box 217, Enschede 7500 AE, The Netherlands; ‡ Sustainable Polymer Chemistry, Faculty of Science & Technology, University of Twente, P.O. Box 217, Enschede 7500 AE, The Netherlands

## Abstract

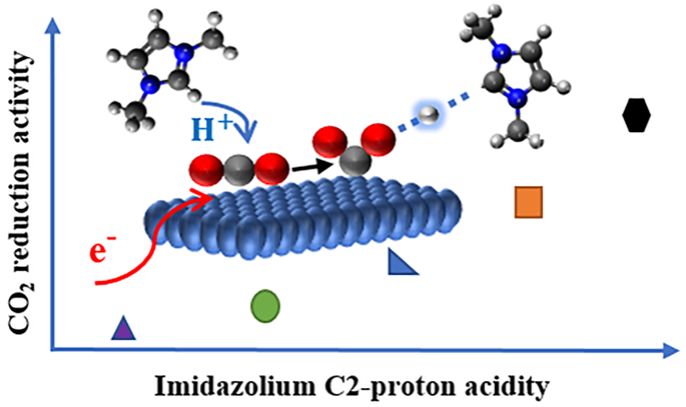

Imidazolium cations enhance the performance of several electrodes in converting CO_2_ to CO in non-aqueous media. In this publication, we elucidate the origin of the function of imidazolium cations when exposed to Au electrodes in anhydrous acetonitrile in CO_2_ atmosphere. We demonstrate that imidazolium cations lead to unprecedentedly low overpotentials for CO_2_ reduction to CO on Au, with ∼100% Faradaic efficiency. By modification of the N_1_ and N_3_ functionality of the imidazolium cation, we show a direct correlation between the performance in CO_2_ reduction and the C_2_–H acidity of the cation. Based on NMR analyses, DFT calculations, and isotopic labeling, showing an inverse kinetic isotope effect, we demonstrate that the mechanism involves a concerted proton–electron transfer to the electrode-adsorbed CO_2_ intermediate. The demonstrated mechanism provides guidelines for improvement in the energy efficiency of non-aqueous electrochemical CO_2_ reduction, by a tailored design of electrolyte cations.

To achieve efficient electrochemical reduction of CO_2_, significant technological advancements in catalyst properties,^1−3^ electrode design (gas diffusion electrodes),^4−6^ and electrolyte composition^7−9^ have recently been reported. However, several challenges still need to be addressed before the technology is commercially attractive,^10,11^ which are particularly associated with the use of aqueous electrolytes. Challenges include the low solubility of CO_2_ in water,^12−14^ the acidification of the electrolyte by dissolution of CO_2_ (including the formation of bicarbonate/carbonate and associated salt precipitation),^15−17^ the competing hydrogen evolution reaction,^18,19^ and finally the instability of metal and metal oxide catalysts in (acid) aqueous environments.^20,21^ The electrochemical inertness of anhydrous solvents, and in particular acetonitrile, resolves most of these disadvantages^12,14,22^—CO_2_ solubility in acetonitrile is about 8 times higher than in water,^23^ and electrode stability against dissolution is also significantly better. However, to promote the electrochemical performance in non-aqueous media, cations need to be added, which both reduce the resistance of acetonitrile solutions and significantly promote conversion of CO_2_. In a variety of experimental conditions, several cations have been demonstrated to be effective in promoting the electrochemical reduction of CO_2_, such as *N*-arylpyridinium salts.^24^ Even more promising is the use of imidazolium salts.^25^ Specifically, Lau et al. employed C_2_-functionalized imidazolium cations to achieve an onset potential of around −2.1 V vs Ag/Ag^+^ for CO_2_ reduction over a Ag electrode in acetonitrile,^26^ and Atifi et al. reported the use of butyl methylimidazolium hexafluorophosphate for conversion of CO_2_ to CO with 85% Faradaic efficiency (FE) using a Bi electrode.^27^ Sung et al. also reported improved efficiency of a molecular Lehn-type catalyst by incorporation of imidazolium species into the secondary coordination sphere.^28^

Despite these efforts, the reported onset potential and associated energy losses in imidazolium solutions remain rather high. Moreover, fundamental knowledge of the function of imidazolium cations is limited, and several hypotheses for the mechanism of promotion have been proposed, including (i) suppression of the H_2_ evolution reaction,^29,30^ (ii) formation of an intermediate imidazolium carboxylate, providing a low-energy pathway toward the conversion of CO_2_ to CO,^31−33^ or (iii) stabilization of the high-energy *CO_2_^®^ intermediate (on Ag surfaces) by hydrogen bonding through the C_4_–H or C_5_–H functionality of C_2_-substituted imidazolium cations.^26^

To discriminate between these hypotheses and properly assess the general function of the cations based on existing literature is difficult, due to the variety of reaction conditions that have been previously applied, including the applied salts (different anions and C_2_-substituted imidazolium cations) and variable water content in solvent compositions.

Here, we selected Au electrodes and anhydrous acetonitrile to systematically investigate how the molecular structure of imidazolium molecules affects the performance in the electrochemical reduction of CO_2_. Since the number of commercially available imidazolium molecules, particularly with a common anion, is limited, we prepared several cations containing C_2_–H functionality, modifying the structure by functionalization of the N_1_ and N_3_ nitrogen. Concerning kinetic improvements in non-aqueous media with the use of imidazolium cations, Ratschmeier and Braunschweig reported an onset potential at ∼ –0.4 V vs SHE for CO formation, albeit in the presence of 10–500 mM H_2_O.^37^ Lau et al. reported onset potentials around −2.0 V vs Fc/Fc^+^ with the use of C_2_-methylated imidazolium cation in acetonitrile.^29^ Here we demonstrate that the systematic chemical modification of the imidazolium cation in combination with anhydrous media leads to an onset potential of around −0.8 V vs Ag/Ag^+^ (∼ –0.258 V vs SHE), which is a significant improvement over previous studies. To the best of our knowledge, this is the lowest overpotential ever reported for reduction of CO_2_ in non-aqueous media. Next, we correlate the performance to the C_2_–H acidity of the imidazolium cation and demonstrate the kinetic relevance of proton donation by isotopic labeling experiments. Finally, DFT calculations complement the study and confirm that a concerted coupled electron–proton transfer mechanism with partial proton transfer is most likely operative.

To ensure similarly low levels of water in anhydrous acetonitrile when comparing the performance of imidazolium cations, we developed an experimental protocol and setup as described in the Experimental Methods. Figure 1a shows linear sweep voltammetry (LSV) for the Au disk electrode immersed in such anhydrous acetonitrile containing 0.5 mol% of 1,3-dimethylimidazolium-NTf_2_ (**MM** NTf_2_) introducing a purge of He or CO_2_ in a small conventional, undivided electrochemical cell. The absence of a noticeable Faradaic current within the potential window of −1.8 to −2.2 V vs Ag/Ag^+^ in the absence of CO_2_ demonstrates the stability of the imidazolium cation against electrochemical conversion, which is also confirmed by NMR analysis. In the presence of CO_2_, significant current can be observed, assigned to the conversion of CO_2_ to CO. Such currents are not observed when other types of cations (cesium-NTf_2_ and tetraethylammonium-NTf_2_) are dissolved in acetonitrile (Figure 1b).

**Figure 1:**
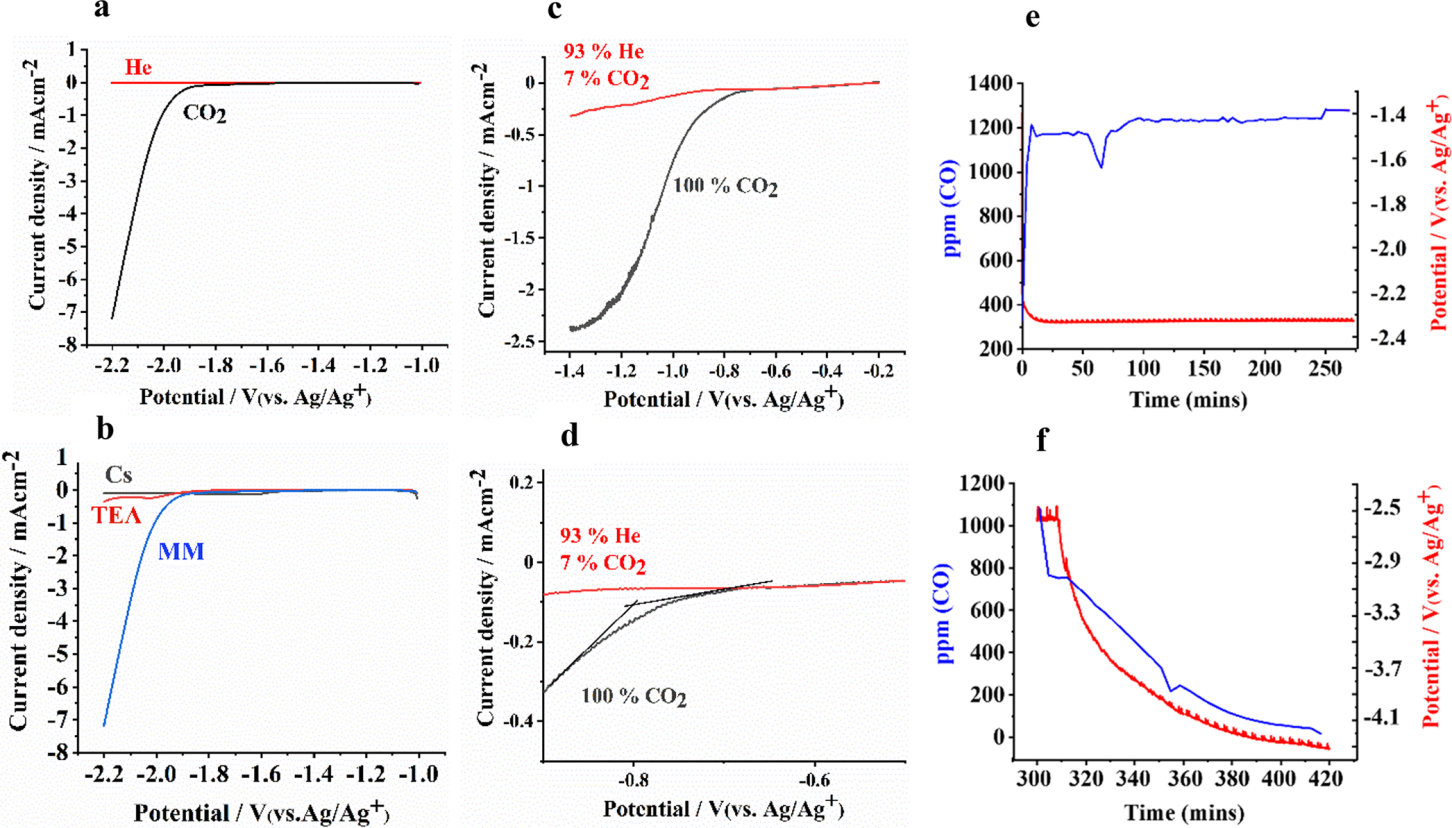
Co-catalytic activity of imidazolium cation for electrochemical reduction of CO_2_ in acetonitrile (<50 ppm water). (a) LSVs with 0.5 mol% of 1,3-dimethylimidazolium (**MM**)–NTf_2_ under He (red) and CO_2_ (black) atmosphere in quiescent solution. (b) LSVs for Au disk electrodes in CO_2_-saturated acetonitrile with dissolved 0.5 mol% of **MM**–NTf_2_ (blue), tetraethylammonium (TEA)-NTf2TEA–NTf_2_ (red), and Cs^+^–NTf_2_ (black). (c) LSVs with 0.5 mol% of **MM** NTf_2_ measured using a rotating Au disk electrode at 2000 rpm: recorded with 7% CO_2_ partial pressure (red) and recorded in CO_2_-saturated acetonitrile (black). (d) Zoomed-in potential region from (c) highlighting the onset potential under CO_2_ purge. (e) Chronopotentiometry at −10 mA/cm^2^ and constant CO production with 0.5 mol% **MM** NTf_2_ under 5 mL/min CO_2_ purge (1.2 bar) and normal stirring conditions using a Au foil electrode (blue), the potential remains stable during electrolysis, and the Faradaic efficiency for CO was 100%. (f) CO (ppm) level measured by GC after switching the flow from CO_2_ to He. The WE potential increases in a similar trend as the concentration of CO is decreasing, due to a decreasing concentration of CO_2_ in the electrolyte as a function of time.

Thus, the unique ability of imidazolium cations in promoting the reduction of CO_2_ in anhydrous media is demonstrated. Performing the experiment in a rotating disc electrode configuration (Figure 1c,d), minimizing mass transfer limitations, demonstrates that the onset potential for CO_2_ activation in anhydrous **MM**-acetonitrile electrolyte is around −0.8 V vs Ag/Ag^+^ (∼ –0.258 V vs SHE (±45 mV), see also Experimental Methods). To the best of our knowledge, this onset potential is the lowest ever reported for activation of CO_2_ in non-aqueous electrolytes.

Chronopotentiometry at −10 mA/cm^2^ shows a high stability in the performance for the production of CO in the **MM**–acetonitrile electrolyte with 100% FE (Figure 1e; for the procedure used to evaluate FE, see the Supporting Information, section III). NMR analyses confirm that, after electrolysis, **MM** NTf_2_ and acetonitrile remain unchanged (see Supporting Information, Figures S33–S35). It should be mentioned that the products formed in the presence of other cations such as tetrabutylammonium-NTf_2_ are mainly hydrogen and methane (Supporting Information, Figure S1), showing that not only activity (on-set potential) but also selectivity (FE) is favorably tuned by the imidazolium cation. Upon switching the CO_2_ flow to He, the concentration of CO decreases gradually while the cell potential increases. Using 10 mL of the **MM**–acetonitrile electrolyte it took ∼90 min to minimize the CO concentration (Figure 1f). The gradual, rather slow, decrease in CO levels after switching the flow of CO_2_ to He is due to the high solubility of CO_2_ in **MM**–acetonitrile electrolyte.^34,35^ As will be discussed in the Experimental Section, the water content was 55 ppm at maximum for all experiments; this water fraction might result in the formation of carboxylate species;^37,40^ however, we did not find any evidence of the presence of such species.

To understand the role of imidazolium cations in promoting CO_2_ reduction in anhydrous conditions, we synthesized various analogs of the **MM** cation and evaluated the performance in the electrochemical reduction of CO_2_ by voltammetry. Figure 2a shows LSVs for **MM**, 1,3-dipropylimidazolium (**n-Pr n-Pr**), 1-propyl-3-isopropylimidazolium (**n-Pr i-Pr**), 1,3-diisopropylimidazolium (**i-Pr i-Pr**), and 1,3-di-*tert*-butylimidazolium (**t-Bu t-Bu**) using a Au electrode in CO_2_-saturated acetonitrile. The structural variation of the imidazolium cation clearly affects the achievable current density. For example at −2 V (vs Ag/Ag^+^), the current density for **t-Bu t-Bu** is lowered by a factor of 10 in comparison to the **MM** analog. Further, chronoamperometry at −1.8 V (vs Ag/Ag^+^) shows that in the presence of 0.5 mol% of **MM**, 3 times more charge is transferred to CO_2_, yielding CO (Figure S2), than achieved with the same amount of **t-Bu t-Bu**.

**Figure 2:**
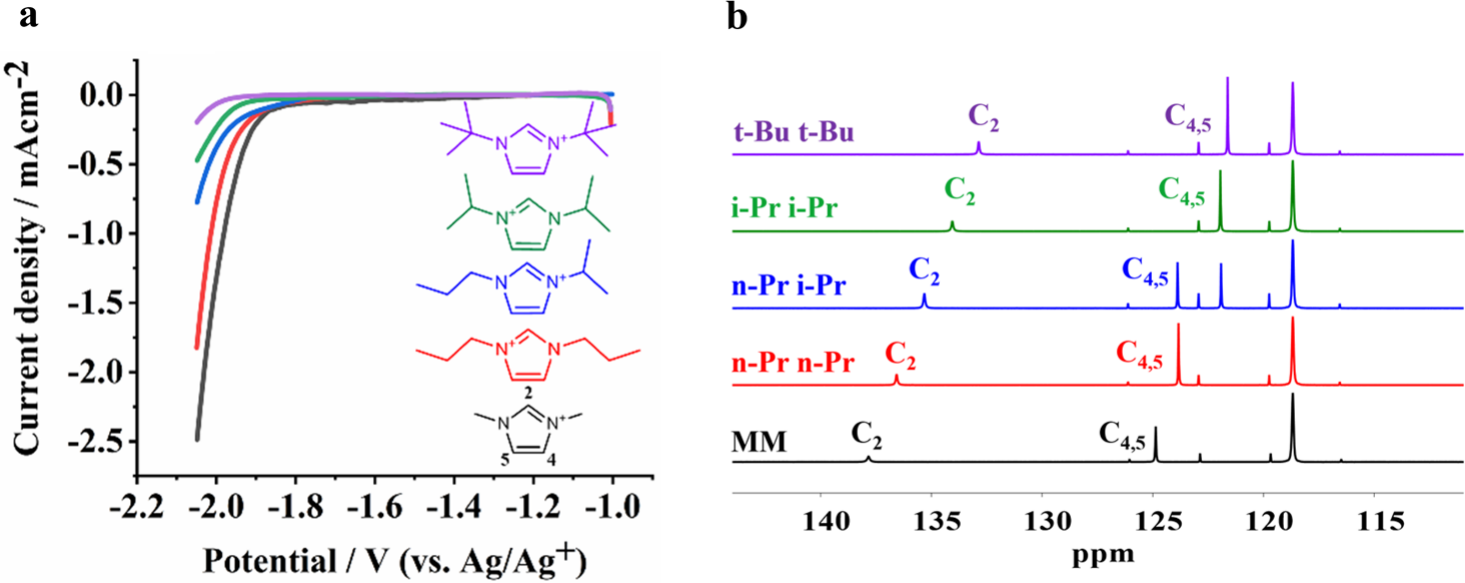
(a) LSVs with 0.5 mol% of 1,3-dimethylimidazolium-NTf_2_ (**MM**), 1,3-dipropylimidazolium-NTf_2_ (**n-Pr n-Pr**), 1-propyl-3-isopropylimidazolium-(**n-Pr i-Pr**), 1,3-diisopropylimidazolium (**i-Pr i-Pr**), and 1,3-di-*tert*-butylimidazolium (**t-Bu t-Bu**) in CO_2_-saturated acetonitrile. (b) Comparison of ^13^C NMR spectra for five cations, recorded in CD_3_CN.

Figure 2b shows ^13^C NMR spectra for five cations. **MM** displays the largest chemical shift (ppm) among all cations for all three carbons of the ring (C2, C4, and C5). Voronoi deformation density (VDD) charge analysis of the cations also shows a more positive charge for the C2 proton and C4, C5 protons of the **MM** cation in comparison to other cations; which is in agreement with the trend obtained in the ^13^C NMR spectra (for VDD charge analysis see Figure S10). The differences in chemical shifts in ^13^C NMR and VDD charge analysis show that the electron density is a function of the substitution, which also affects the C2–H acidity. p*K*_a_ calculations/measurements in acetonitrile from previous studies also demonstrate a trend in the acidity for **MM** (32.5), **i-Pr i-Pr** (33.6), and **t-Bu t-Bu** (34.1) cations,^36,37^ which is in agreement with the trend obtained with ^13^C NMR spectra (Table S1). Interestingly, and most importantly, the electron density of the imidazolium cation translates to higher or lower performance in electrochemical reduction of CO_2_, showing an almost linear trend between ^13^C peak position (Figure 2b) and current density in the reduction of CO_2_, as schematically indicated in the graphical abstract of this paper.

With this structure–activity relationship established, we can now address the question how imidazolium promotes CO_2_ reduction in anhydrous media. We will first compare the significance of the C2 proton with that of the C4 and C5 protons of the imidazolium ring. In the literature discussing electrochemical CO_2_ reduction, the first electron transfer (from the electrode to adsorbed CO_2_) is well known to be the rate-determining step (RDS).^10,38^ Based on the literature, we assume that the surface of the Au electrode promotes the initial electron transfer to CO_2_, to form a high-energy *CO_2_^–^ intermediate.^39,40^ Our DFT calculations in acetonitrile show that there is a ∼70 kcal/mol difference in free energy for adsorbed *CO_2_^–^ (Au-CO_2_^–^) versus the solved CO_2_^–•^ radical (CO_2_^–•^(sol)), confirming the hypothesis of adsorption-induced electron transfer (* + CO_2_ + e^–^ = *CO_2_^–^) in acetonitrile (Supporting Information, section VI). The structure–activity relationship developed in this work suggests that ring protons H2, H4, and H5 might be involved in promoting the kinetics by stabilizing the *CO_2_^–^ intermediate. While the trend in NMR chemical shifts for C2 is more obvious than for C4 or C5 (Figure 2b), it remains inconclusive which ring proton is involved in stabilizing the *CO_2_^®^ intermediate. We employed DFT calculations to compare the stability of *CO_2_^®^ when brought into interaction with the C2 proton (*configuration i*) or C4 and C5 protons (*configuration ii*) of the **MM** cation (Figure 3). Four kcal/mol is gained for *configuration i* versus *configuration ii*. Surface charge density and differential VDD charge analysis (Figure 3, bottom part; also see Supporting Information, Figure S12) show that with the C2 proton *configuration i*, a more uniform charge distribution is obtained (please note the red and blue color densities). To further investigate the mode of operation of the C2 proton, the activity of **MM** was compared to that of 2-methylated MM (**2-Me MM**) (Supporting Information, Figure S3). The substantial decrease in the current density observed for **2-Me MM** compared to **MM** suggests that the C2 proton is most effective in promoting the reduction of CO_2_.

**Figure 3:**
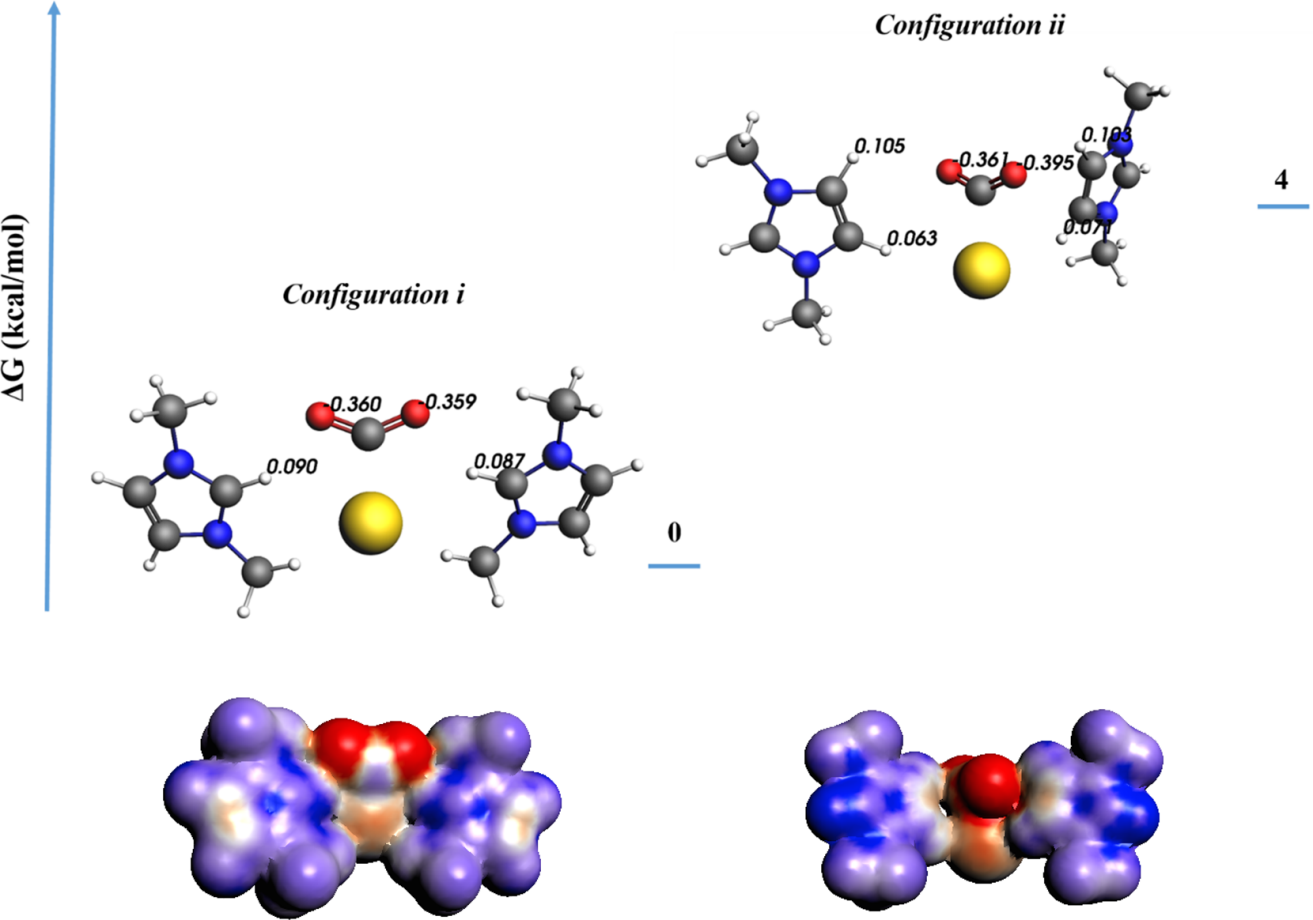
4 kcal/mol energy is gained when the C_2_ proton is brought into interaction with *CO_2_^–^(*configuration i* vs *configuration ii*). This extra energy gain can be understood in terms of a more uniform charge distribution. VDD charges are shown for the oxygen atoms of *CO_2_^–^ and involved C_2_, C_4_, and C_5_ protons (for differential charge analysis see Supporting Information, Figure S12). Surface charge densities (on COSMO surface) are constructed and are depicted below each relevant configuration.

We will now investigate how the C_2_ proton affects the reduction of CO_2_. *Pathway i* (Figure 4a) considers electron transfer to the cation to form a cation radical as proposed by Wang et al.^31^ For this pathway, the reduced cation acts as an initiator and reduces CO_2_ through a nucleophilic attack to form an imidazolium–CO_2_ adduct (Supporting Information, Scheme S1). After the nucleophilic attack, the C_2_ proton is transferred via an isomerization step, which is considered the rate-determining step (RDS). However, DFT calculations for the isomerization step do not show any noticeable difference in the activation energy for several selected cations (Supporting Information, section VIII and Figures S14 and S15). Besides, *pathway i* implies an electrochemical response in the absence of CO_2_ (the electron transfer to the imidazolium cation), which is not observed in Figure 1. If an initial electron transfer to the imidazolium cation were the case, the same onset potentials would be observed for the reduction of the cation (CVs under He purging) and reduction of CO_2_ (CVs under CO_2_ purging). This situation is typical when pyridinium-type molecules are used as the promoter, but this is not the case with imidazolium cations. (For more arguments, see the Supporting Information, section IX and Figure S16).

**Figure 4:**
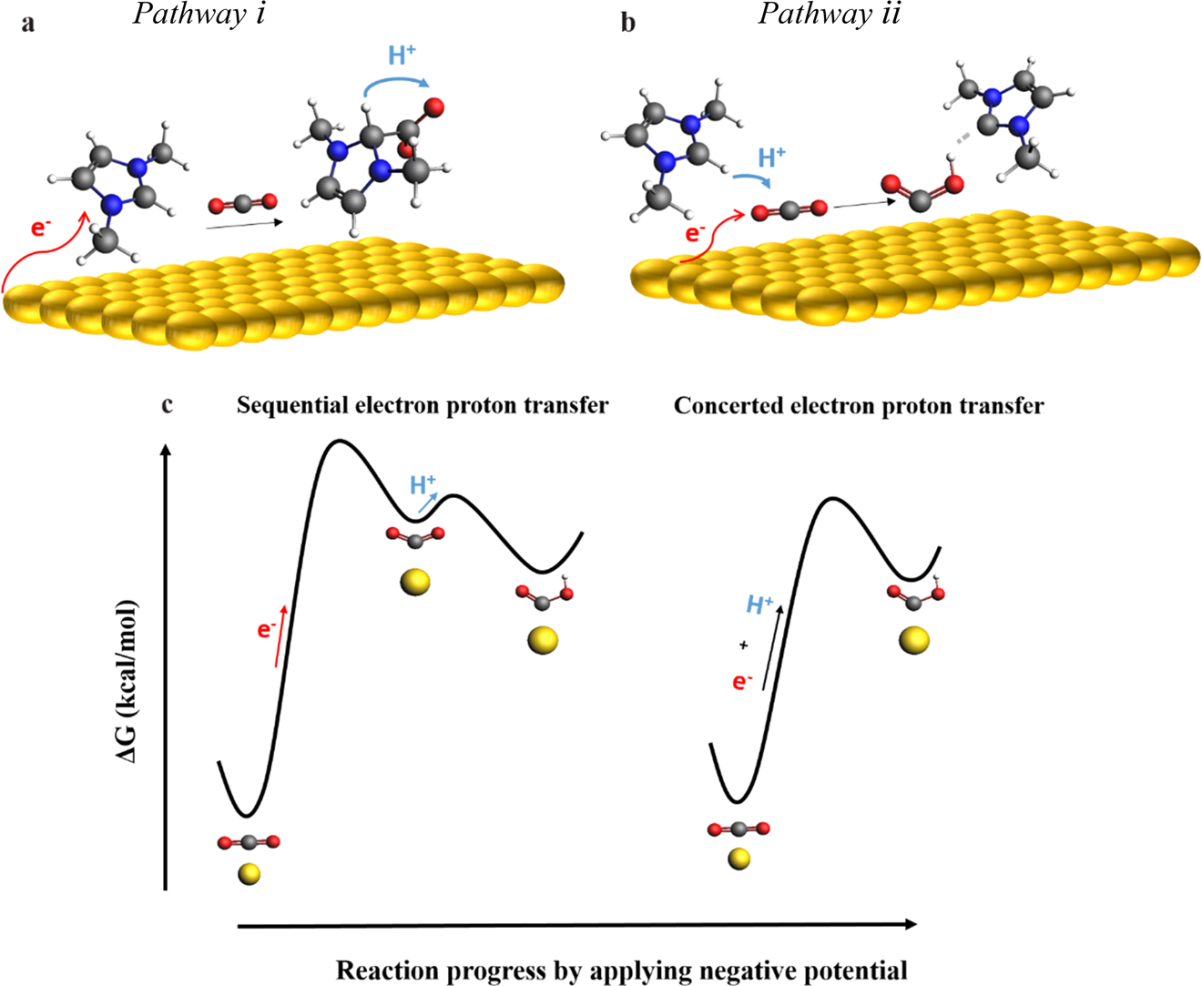
(a) *Pathway i* depicts an initial electron transfer to the **MM** cation followed by a sequential PT-ET to CO_2_. (b) *Pathway ii* (proposed in this work) depicts the concerted mechanism in which a bidirectional ET (form Au electrode) and PT (from C_2_ proton of **MM** cation) leads to a lower energy barrier. (c) Schematic of the energy diagram for stepwise and concerted pathways.

Thus, even though the mechanism proposed in *pathway i* might be considered in electron transfer reactions in homogeneous solution, Au catalyzed surface transformations appear to follow another mechanism. *Pathway ii* (Figure 4b) depicts the initial electron transfer to CO_2_ upon its adsorption. Upon electron transfer, the C2 proton of the imidazolium cation interacts with the negatively charged *CO_2_^®^ being formed on the Au surface. Such a charge transfer is also confirmed by differential charge analysis from the interaction between **MM** and *CO_2_^®^ (Figure 3, and Supporting Information, Figure S12). This pathway highlights a concerted coupled electron–proton transfer (CEPT) mechanism. For this mechanism, the first electron transfer should still be rate-determining, and this was confirmed with the obtained Tafel slope of 119 mV/dec (Supporting Information, Figure S4). CEPT reactions are of significant importance because they can bypass high-energy intermediates present in sequential ET and PT steps and generally occur with lower reaction barriers (Figure 4c).^41−43^

For a CEPT mechanism, an *(OC)O···H intermediate develops during the rate-determining step. To determine the kinetic significance of the *(OC)O···H intermediate, we first calculated the difference in frequencies between O–H and deuterated O–D of the adsorbed intermediate (Δσ)_In_, to be 964 cm^–1^ (in these calculations, complete covalent O–H and O–D bond formation were considered), which is larger than the difference in frequency of the C–H and C–D bonds in the imidazolium cations ((Δσ)_Gs_ = 837 cm^–1^) (see Supporting Information, section XIX). This difference in differential frequencies leads to a larger difference in the ΔZPE (zero-point energy) of the transition state than in the ΔZPE of the ground state, suggesting an inverse kinetic isotope effect (iKIE, *k*_H_/*k*_D_ = 0.735), when a covalent bond character is considered (see Figure 5a). Thus, from frequency calculations, it is predicted that for a CEPT mechanism, deuterium substitution at the C_2_ position of the imidazolium cation should result in a higher current density (lower activation energy) in the reduction of CO_2_. The inverse kinetic isotope effect was indeed experimentally identified when performing the reduction of CO_2_ in an RDE setup (*k*_H_/*k*_D_ = 0.89; Figure 5b and Supporting Information, Figure S5). The deuterated version of **MM** shows significantly larger current densities, with an 18% deviation between the calculated and experimentally determined iKIE. This deviation can be rationalized in the context of a partial proton transfer in the concerted mechanism (see also Supporting Information, section XI, for extra notes on iKIE) and that there is still an opportunity to further improve performance by optimizing the *(OC)O^δ−^···^δ+^H–C2 bond in the transition state, lowering the energy of the transition state.

**Figure 5:**
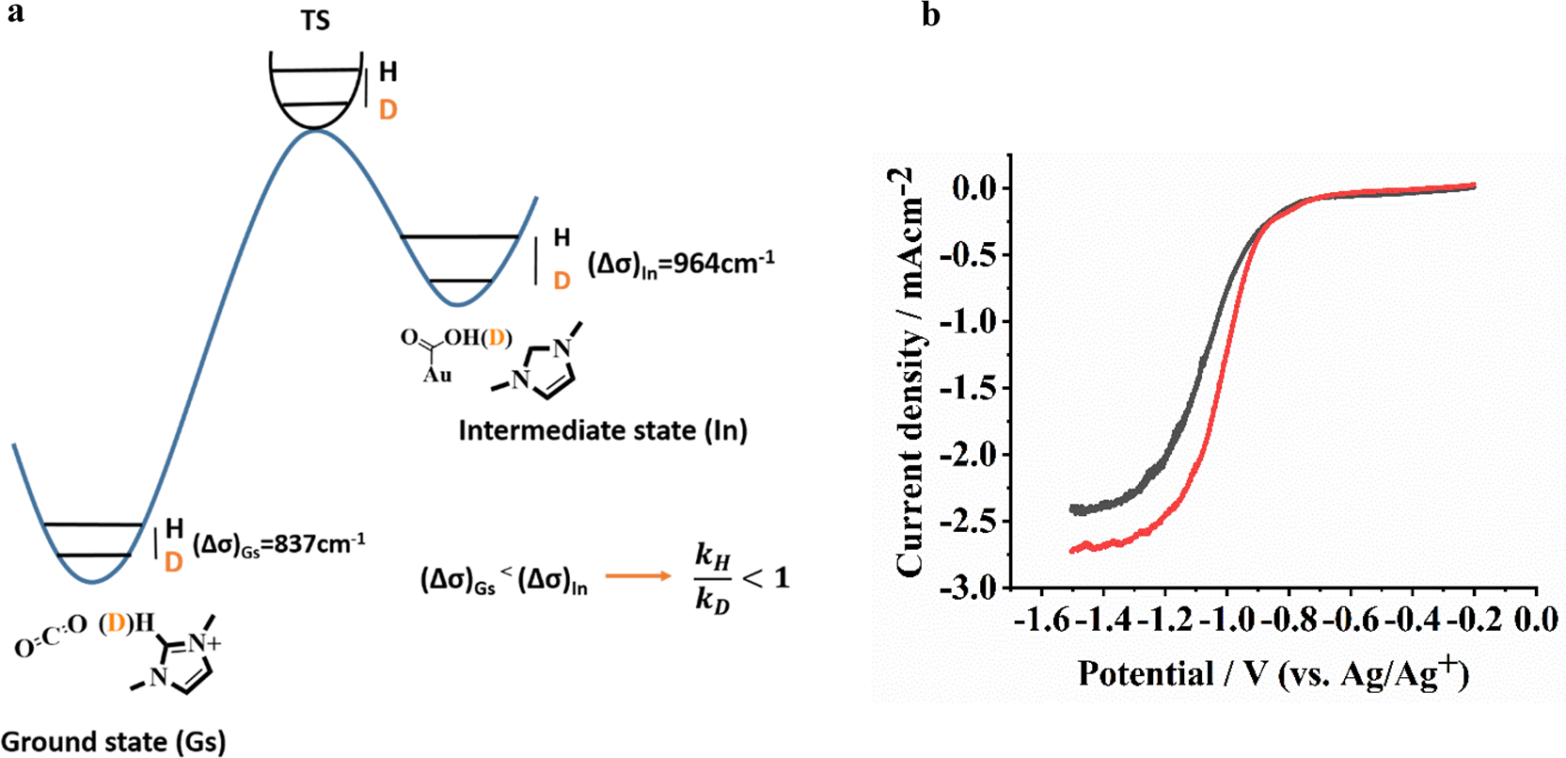
(a) DFT calculation for a CEPT rate-determining step for **MM** co-catalyzed CO_2_ reduction in anhydrous acetonitrile. (b) LSVs recorded using Au rotating disk electrode (2000 rpm) with **MM** and **deuterated MM** in CO_2_-saturated acetonitrile showing an experimental inverse kinetic isotope effect, For quantification of iKIE, see Supporting Information, Figure S5.

In summary, in this study, we have demonstrated a correlation between the C_2_–H acidity of imidazolium cations and achievable current density in the reduction of CO_2_ in anhydrous conditions, by changing the functionality of the molecule at the N_1_ and N_3_ positions. The inverse kinetic isotope effect and Tafel analysis show that the rate-determining step involves a concerted coupled electron–proton transfer with a partial proton transfer, which leaves room for even further optimizing the molecular structure. What we have not assessed in the present study, is the interaction of the conjugated base of the imidazolium cation (carbene) with the electrode. *N*-Heterocyclic carbenes (NHCs) readily bind transition metals by σ-donation,^44^ and recently their great affinity for electrode surfaces such as Au has been shown.^45^ The relevance of such interaction for performance in the electrochemical reduction of CO_2_, needs to be assessed for electrodes of different binding energy. Another intriguing aspect of the undivided cell is the overall electrochemical process at play, including the anodic reaction. Additional work is required to understand the closure of the electrochemical cycle, as discussed in the Supporting Information, section XII. Whatever the overall reaction, one could purposely add a chemical to the solvent, which can be oxidized and allows so-called paired electrolysis. An eye-catching feature of anhydrous **MM**–acetonitrile versus aqueous media is the broad electrochemical window recorded under He purging (Supporting Information, Figure S6), extending over 5 V (!) (vs 2.2 V for aqueous **MM**–water), allowing, for example, the electrosynthesis of acetophenone and the oxidative dimerization of stilbenes.^46−48^ Anhydrous imidazolium–acetonitrile electrolyte is thus quite promising for designing the desired electrochemical catalytic cycle, and particularly the simple structure–activity relation developed in this work brings up opportunities to increase the efficiency even further by molecular design of cations.

## Experimental Methods

### Preparing Anhydrous Conditions

All glassware and reactors were rinsed with Milli-Q water (Milli-Q Reference, 18.2 MΩ.cm, TOC value below 5 ppb, Merck), ethanol, and HPLC-grade acetonitrile (99.9%, Sigma-Aldrich) before electrochemical measurements. For every measurement, the voltammetry electrode was first rinsed with Milli-Q water and ethanol. An aluminum polishing pad (Prosense, QVMF 1040) was wetted with ethanol, and the electrode was gently polished for 4 min (no alumina was used over the polishing pad). After sonication in 0.5 molar HNO_3_, the electrode was electrochemically cleaned in 0.1 M H_2_SO_4_. For electrochemical cleaning, CVs were recorded first at 20 cycles with 1 V/s scan rate followed by 10 cycles at 100 mV/s (0.2 to 1.5 V vs Ag/AgCl). After 10 min sonication in Milli-Q water, the electrode and all other parts of the reactor (reference compartment, graphite rod and gas inlet, and gas outlet tubes) were assembled and were sonicated for 2–5 min with anhydrous acetonitrile. After assembling, the reactor was kept under a purge of super dry He (Helium A/Zero grade N4.6, Linde) for 30 min. Solutions were transferred with caution into the reactor with gastight syringes. A dryer (ZPure DS H2O, ChromRes) was used to further remove any moisture from the CO_2_ inlet (Carbon Dioxide Food grade, Linde) before entering the reactor. Before solution injection, the reactor was washed twice with anhydrous acetonitrile (transferred from a nitrogen-filled glovebox). After injection, the solutions were kept under He/CO_2_ purge for 1 h to remove any remaining oxygen. Karl Fischer titrations were performed to measure the water content from solutions inside the glovebox and after the injection into the reactor. ∼48–55 ppm water was found for all solutions, and it was confirmed that no water was introduced to the solutions upon transfer from the glovebox into the reactor.

### Reference Electrode Preparation

In order to avoid the cross-contamination of the working solution with other organic cations, for each cation, a new reference solution was prepared for the electrochemical measurement. Reference solutions always contained 0.1 mol% (0.02 M) of Ag-OTf with 0.4 mol% of the electrolyte subject to study (in total 0.5 mol% of salt concentration in anhydrous acetonitrile). The reference solution was separated from the working solution by an ultrafine frit. Ag wire was used as the pseudoreference electrode. The potential recorded versus the Ag reference electrode immersed in an electrolyte containing 0.02 M silver salt in acetonitrile can be converted to the SHE scale by the following equation:^49^
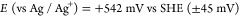


### Electrochemical Measurements

Working solutions always contained 0.5 mol% of the imidazolium cation and were all prepared inside the glovebox. NTf_2_ was always used as the common electrolyte anion, and anhydrous acetonitrile was the common solvent for all measurements. A graphite rod (99.99% Sigma-Aldrich) was used as the counter electrode. Gas chromatography (Compact GC 4.0, Interscience) was used to analyze the gas products from the reactor. Helium was used as the carrier gas, and the GC was calibrated for 1 to 100,000 ppm of CO (Carbon Monoxide CP grade N3.0, Linde). All electrochemical measurements were performed with a Biologic SP-300 potentiostat in a three-electrode configuration. RDE measurements were performed using a WaveVortex 10 Electrode Rotator (Pine Research). For all measurements, the flow of the gas inlet was 5 mL/min, and LSVs were recorded in quiescent solution to maintain the same hydrodynamic conditions. Before any electrochemical measurement, we made sure that a stable open-circuit voltage was established. For all measurements, reproducibility was checked (see Supporting Information, Figure S7). For evaluation of FEs and GC analysis see Supporting Information, Figures S8 and S9.

### Synthetic Procedure

For full synthetic details and NMR spectra of the as-synthesized compounds, some before and after reaction, see the Supporting Information, section XII and Figures S17–S38.

### Characterization

400 MHz ^1^H, 100 MHz ^13^C, and 376 MHz ^19^F NMR spectra were recorded on a Bruker Avance III 400 NMR spectrometer in CD_3_CN. Solvent residual signals with chemical shifts of 1.94 ppm (^1^H NMR) and 118.69 ppm (^13^C NMR) were used as references. Full spectra are provided in the Supporting Information.

### Computational Methods

DFT calculations were performed with the Amsterdam density functional program.^50^ B3LYP was used as a hybrid exchange-correlation functional^51^ together with Grimme’s DFT-D3 dispersion correction with Becke–Johnson (BJ) damping.^52^ For all atoms, a Slater-type basis set of triple-ζ valence quality with two polarization functions was used (TZ2P).^53^ For all calculations, scalar relativistic effects using the zeroth-order regular approximation (ZORA) formalism were included. For calculations including gold atoms, spin–orbit coupling was considered. Calculations were always performed with no frozen core (an all-electron basis set) with a “very good” numerical quality. All calculations were performed with a conductor-like screening model (COSMO) to account for solvent effects (acetonitrile). Results of calculation and detailed descriptions are provided in the Supporting Information (Figures S10–S13 and Table S1).

## Notes

Frequencies for transition states and for compounds relevant to kinetic isotope effect and raw data are available from the corresponding author on reasonable request.

## Supporting Information Available

Materials, electrochemical data, results from DFT calculations for mechanistic studies, methods for evaluating kinetic isotope effects, ^1^H NMR, ^13^C NMR, and ^19^F NMR spectra, and gas analysis data, including Figures S1–S38, Scheme S1, and Tables S1 and S2 (PDF)
