# Supplementary material for: What It Takes for Imidazolium Cations to Promote Electrochemical Reduction of CO2
Source: ACS Energy Lett. 2022 Sep 15;7(10):3439–46. doi: 10.1021/acsenergylett.2c01372 (PMC9578038; doi:10.1021/acsenergylett.2c01372)
Supplement: Supplementary file 1 — nz2c01372_si_001.pdf [file nz2c01372_si_001.pdf]

Supporting Information

What it takes for imidazolium cations to promote electrochemical reduction of CO_2

Sobhan Neyrizi[1,2], Joep Kiewiet[1], Mark A. Hempenius[2], and Guido Mul[1]*

[*]Corresponding Author: g.mul@utwente.nl

[1]Photocatalytic Synthesis Group, Faculty of Science & Technology of the University of Twente, PO Box 217, Enschede, The Netherlands.

[2]Sustainable Polymer Chemistry, Faculty of Science & Technology of the University of Twente, PO Box 217, Enschede, The Netherlands.

Table of Contents

I. **Materials**

II. **Electrochemical data**

III. **GC analysis and Faradaic efficiencies**

IV. **VDD charge analysis of cations**

V. **^{13}C NMR chemical shifts (ppm) of imidazolium NTf2 salts in CD3CN**

VI. **Stability of $^{*}CO_2^{-}$ vs free CO_2 anion radical in acetonitrile**

VII. **Interaction of C2-proton and C4, C5-protons of MM with Au-CO_2^{-}**

VIII. **Activation energy analysis for isomerization step**

IX. **Notes on the initial act of the electrode**

X. **Inverse kinetic isotope effect estimated from frequency calculations**

XI. **Extra notes on inverse kinetic isotope effect**

XII. **Notes on the electrochemical cycle**

XIII. **Synthetic procedures**

XIV. **Copies of ^{1}H, ^{13}C, and ^{19}F NMR spectra**

XV. **References**

I. Materials

Gold (Au) voltammetry electrodes (3.0 mm diameter, 99.95%) were purchased from Prosense. Gold wire (0.025 mm diameter, 99.99 %) for electrolysis experiments were supplied by Sigma Aldrich. Anhydrous acetonitrile (99.8%), acetonitrile (ReagentPlus, 99%), dichloromethane (puriss p.a. ACS reagent ≥99.9%), diethyl ether (anhydrous, ACS reagent, ≥99.0%), tetraethylammonium bis(trifluoromethylsulfonyl)imide (97%) and tetrabutylammonium bis(trifluoromethylsulfonyl)imide (99%), bis(trifluoromethane)sulfonimide lithium salt (99%), silver trifluoromethanesulfonate (≥99%), deuterium oxide (99.9 atom % D), acetonitrile-d_3 (≥99.8 atom % D), 1,3-diisopropylimidazolium chloride (97%) and 1,3-di-*tert*-butylimidazolium tetrafluoroborate (97%) were obtained from Sigma-Aldrich. Acetonitrile-d_3 (99.8 atom % D) for electrolysis experiments, Cs bis(trifluoromethylsulfonyl)imide (98%) , and 1-iodopropane (99%) were obtained from Acros Organics. 1-Isopropylimidazole (98%) was obtained from abcr GmbH, Karlsruhe. 1,3-Dimethylimidazolium bis(trifluoromethylsulfonyl)imide (99%), 1-methyl-3-pentyl imidazolium bis(trifluoromethylsulfonyl)imide (99%), and 1,3-dipropylimidazolium bis(trifluoromethylsulfonyl)imide (98%) were purchased from Iolitec Ionic Liquids Technologies GmbH. Milli-Q water was taken from a Milli-Q Advantage A10 Water Purification System, Millipore (18 MΩ.cm). 1,3-Di-*tert*-butylimidazolium tetrafluoroborate and 1,3-diisopropylimidazolium chloride were anion exchanged for NTf_2 and were transferred to the glove box after vacuum drying.

II. Electrochemical data

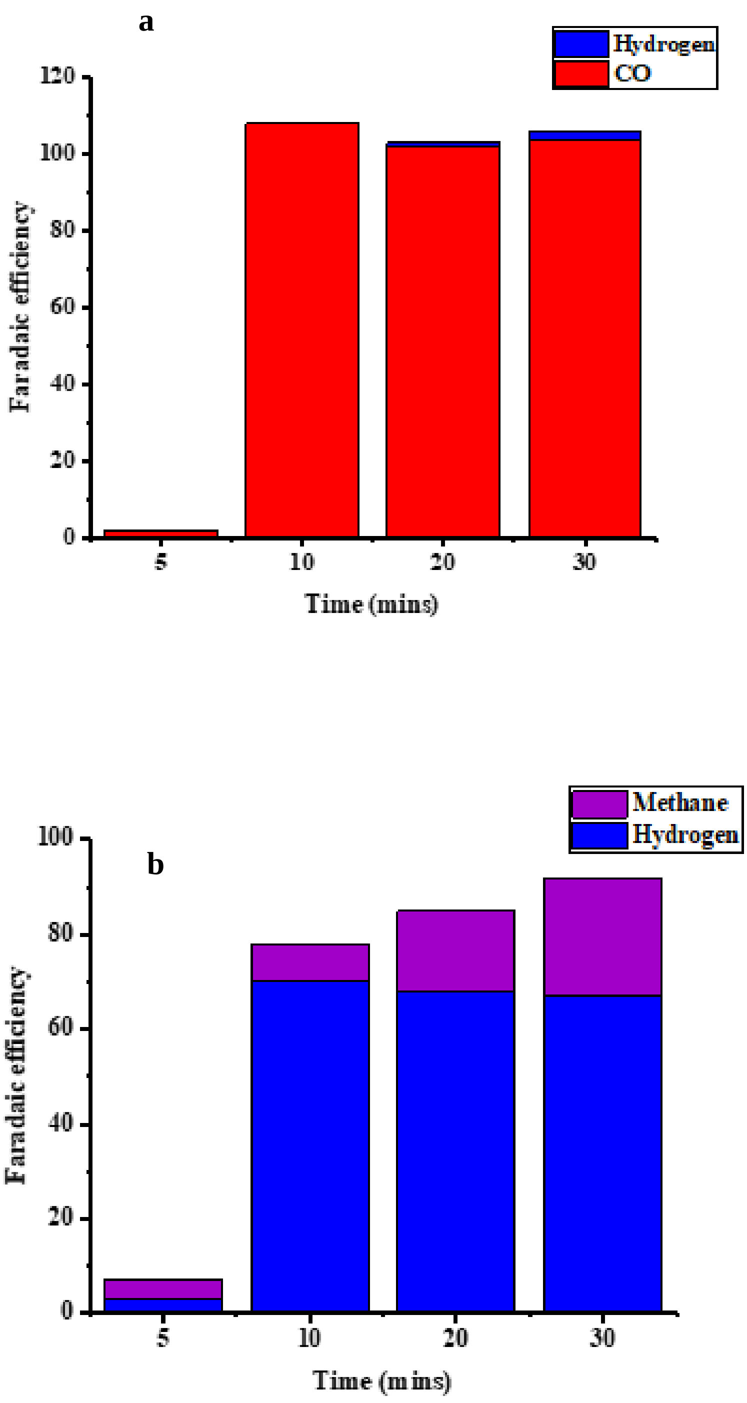

Figure S1. Comparison of the performance for CO_2 reduction with 1.5 mol% of 1-methyl-3-pentyl imidazolium (PM) and 1.5 mol% of tetrabutylammonium (TBA) cations in anhydrous acetonitrile. a, Electrolysis with PM NTf_2 at 18 mA/cm^2 shows ~100 % FE for CO production at an average voltage of -2.58 V vs. Ag/Ag^+. **b,** Electrolysis with TBA NTf_2 at 18 mA/cm^2 results in lower performance for both selectivity and productivity: H_2 was the main product with an average voltage of -3.3 V vs. Ag/Ag^+. Electrolysis measurements were performed over the Au foil electrode in a two-compartment cell under normal stirring conditions and applying a flow of 5 mL CO_2/min.

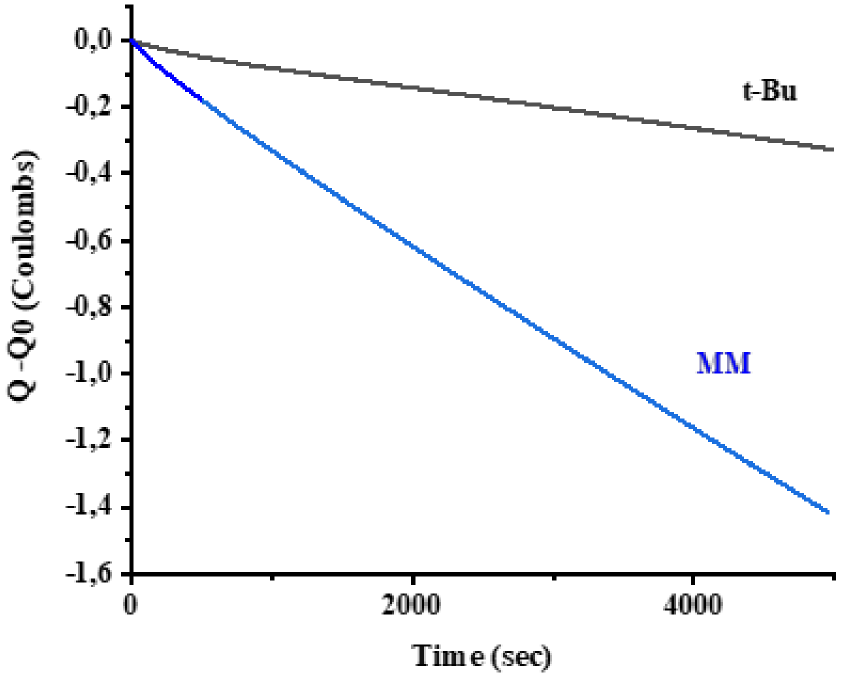

Figure S2. Comparisons of the stable electrochemical activity of MM and t-Bu for CO_2 reduction. The charges transferred in the presence of 0.5 molar % of **MM** NTf_2 and 0.5 molar % of **t-Bu** NTf_2. The potential was constant at -1.8 V vs. Ag/Ag^+, and the reactor was purged with 5 mL/min CO_2. The stability of **t-Bu** NTf_2 was also checked after chronoamperometry (Figures S36 to S38).

$$\frac{(\Delta q_{CO_2} - \Delta q_{He})\,\mathrm{MM}}{(\Delta q_{CO_2} - \Delta q_{He})t-Bu} = \frac{-0.53203\ coulombs/hr}{-0.16742\ coulombs/hr} = 3.177$$

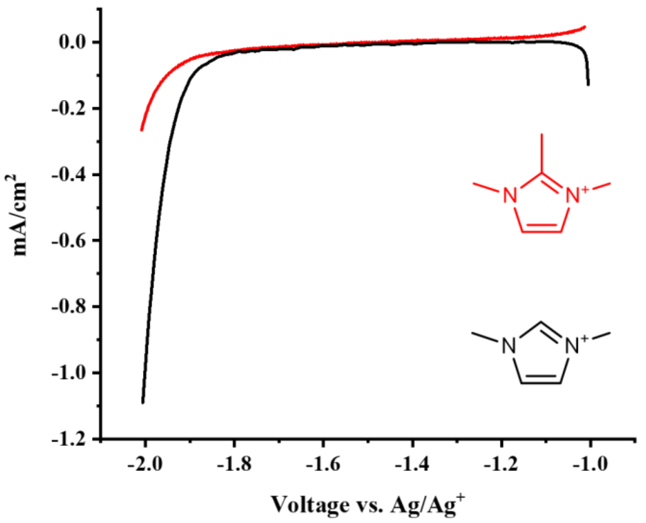

Figure S3. Importance of the C2-proton in determining CO_2 reduction activity. Comparison of CO_2 reduction activity for the **MM** cation with that of the **2-methylated MM** cation. LSVs were recorded in CO_2 saturated acetonitrile at an Au disk electrode.

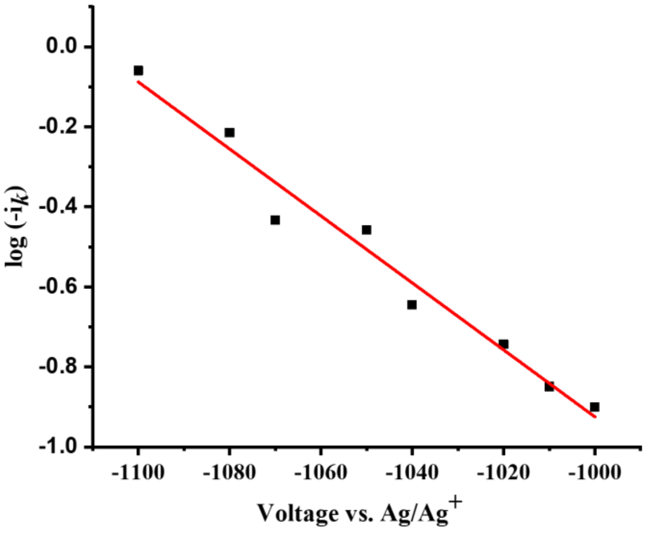

Figure S4. Tafel analysis for CO_2 reduction in anhydrous MM-acetonitrile. The Koutecky-Levich equation was used to obtain kinetic currents (-*ik* in mA) within the linear region of the current-potential profile using the RDE set up at different rpms.

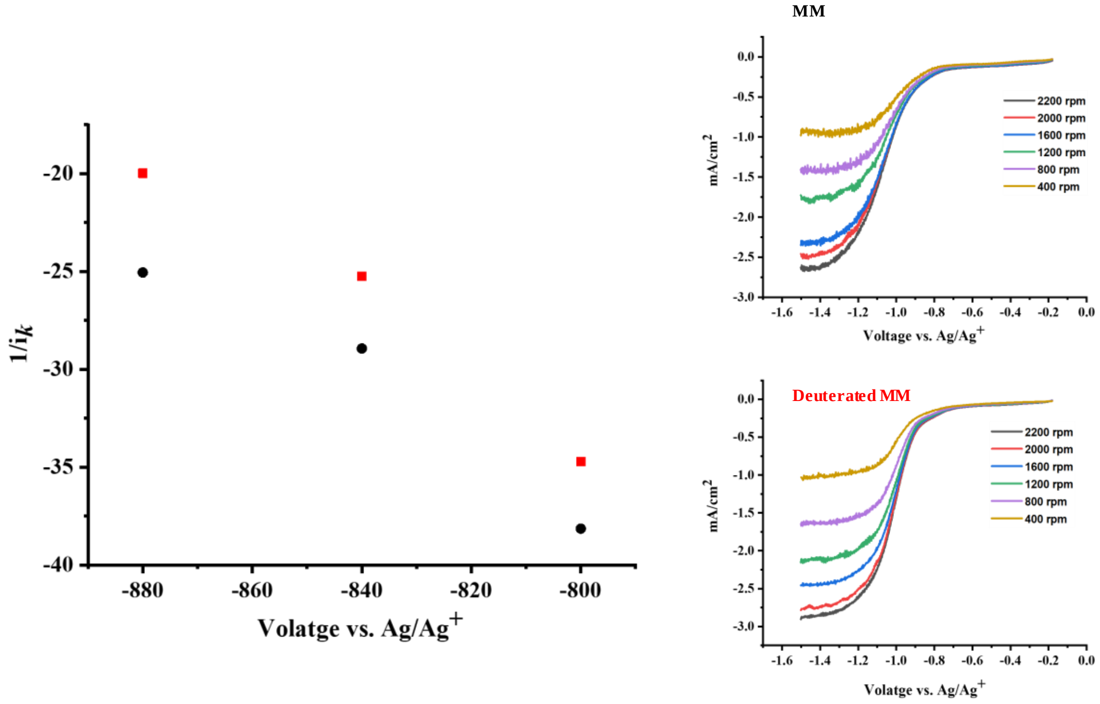

Figure S5. The determination of the inverse kinetic isotope effect for CO_2 reduction in anhydrous imidazolium-acetonitrile. The Koutecky-Levich equation was used to obtain kinetic currents (*ik* in mA) for both **MM** and **deuterated MM** with the RDE set up at different rpms (right). Kinetic currents at low overpotentials were used to evaluate the inverse kinetic isotope effect (left).

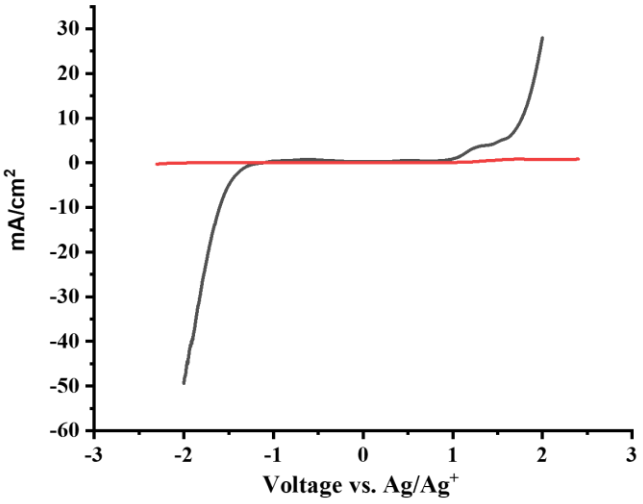

Figure S6. Electrochemical window of MM-acetonitrile vs MM-water. LSVs for Au disk electrode under the purge of He in water with 0.5 mol% of **MM** Cl (**red**) and in acetonitrile with 0.5 mol% of **MM** NTf_2.

For all LSV measurements, the experiments were repeated to determine reproducibility. For each cation two LSVs are compared as follows.

MM

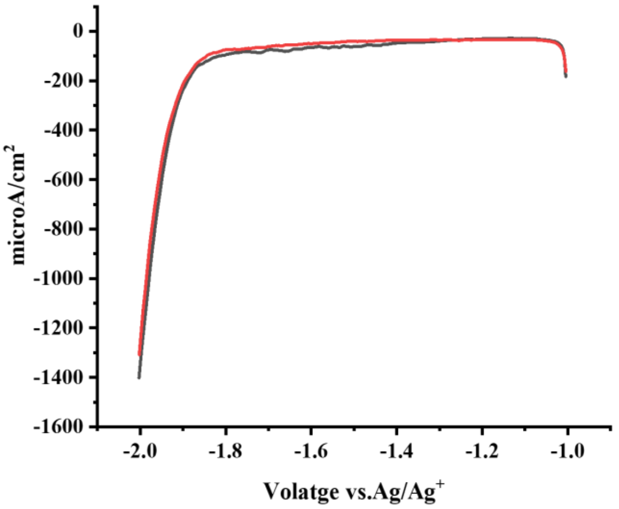

n-Pr n-Pr

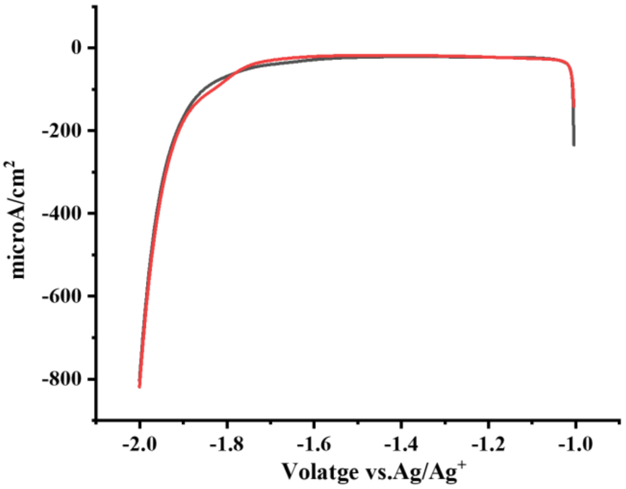

Figure S7 a. Reproducibility of LSVs for the activity of imidazolium cations for CO_2 reduction in anhydrous acetonitrile. LSVs with 0.5 mol% of imidazolium salts in CO_2 saturated anhydrous acetonitrile at Au disk electrode.

n-Pr i-Pr

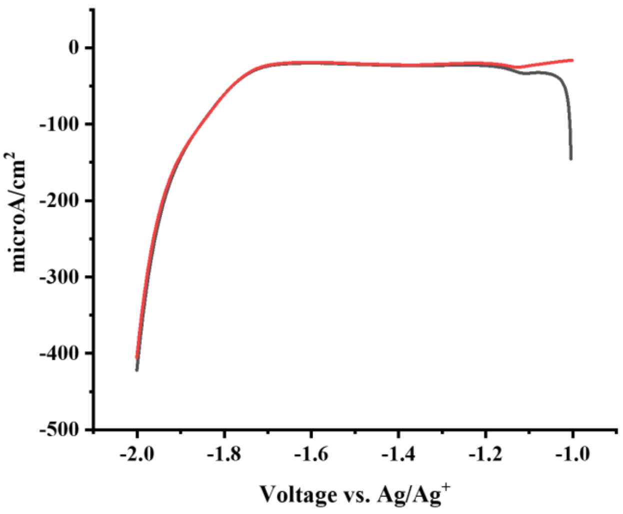

i-Pr i-Pr

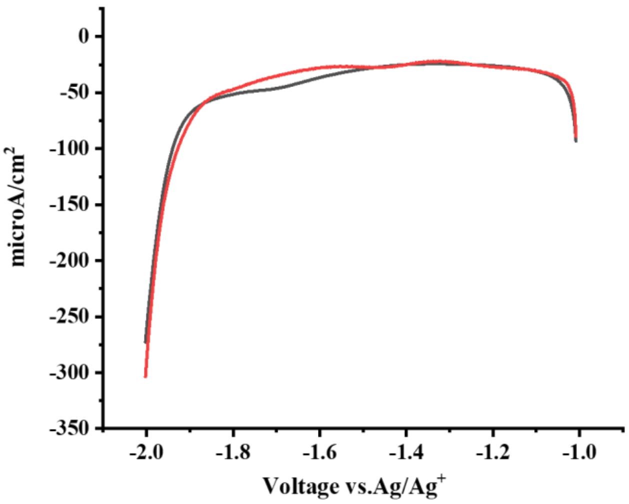

Figure S7 b. Reproducibility of LSVs for the activity of imidazolium cations for CO_2 reduction in anhydrous acetonitrile. LSVs with 0.5 mol% of imidazolium salts in CO_2 saturated anhydrous acetonitrile at Au disk electrode.

t-Bu t-Bu

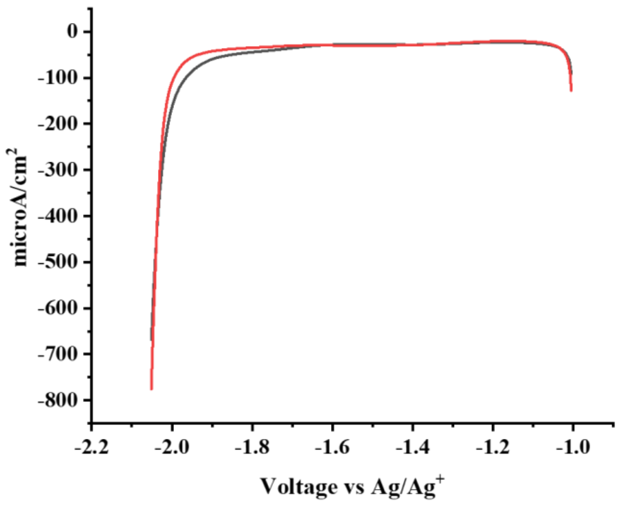

2-Me MM

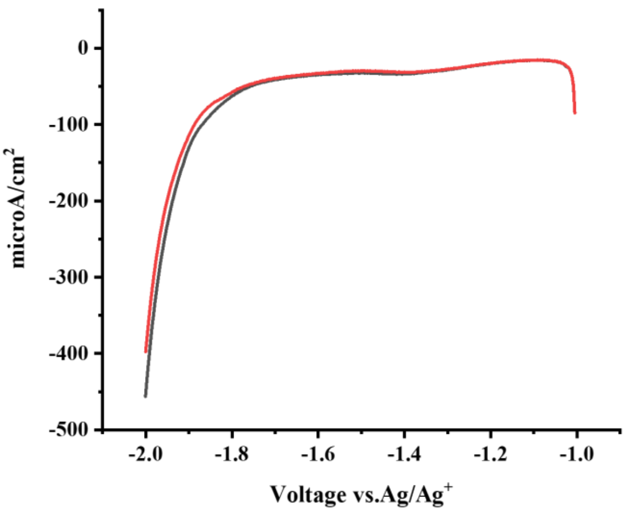

Figure S7 c. Reproducibility of LSVs for the activity of imidazolium cations for CO_2 reduction in anhydrous acetonitrile. LSVs with 0.5 mol% of imidazolium salts in CO_2 saturated anhydrous acetonitrile at Au disk electrode.

III. GC analysis and Faradaic efficiencies

To evaluate Faradaic efficiency for a reaction, gas and liquid products were analyzed. For gas analysis, GC was first calibrated for the relevant ppm's of CO and other potential products for CO_2 reduction. Figure S8 shows examples of the calibration data for CO and H_2.

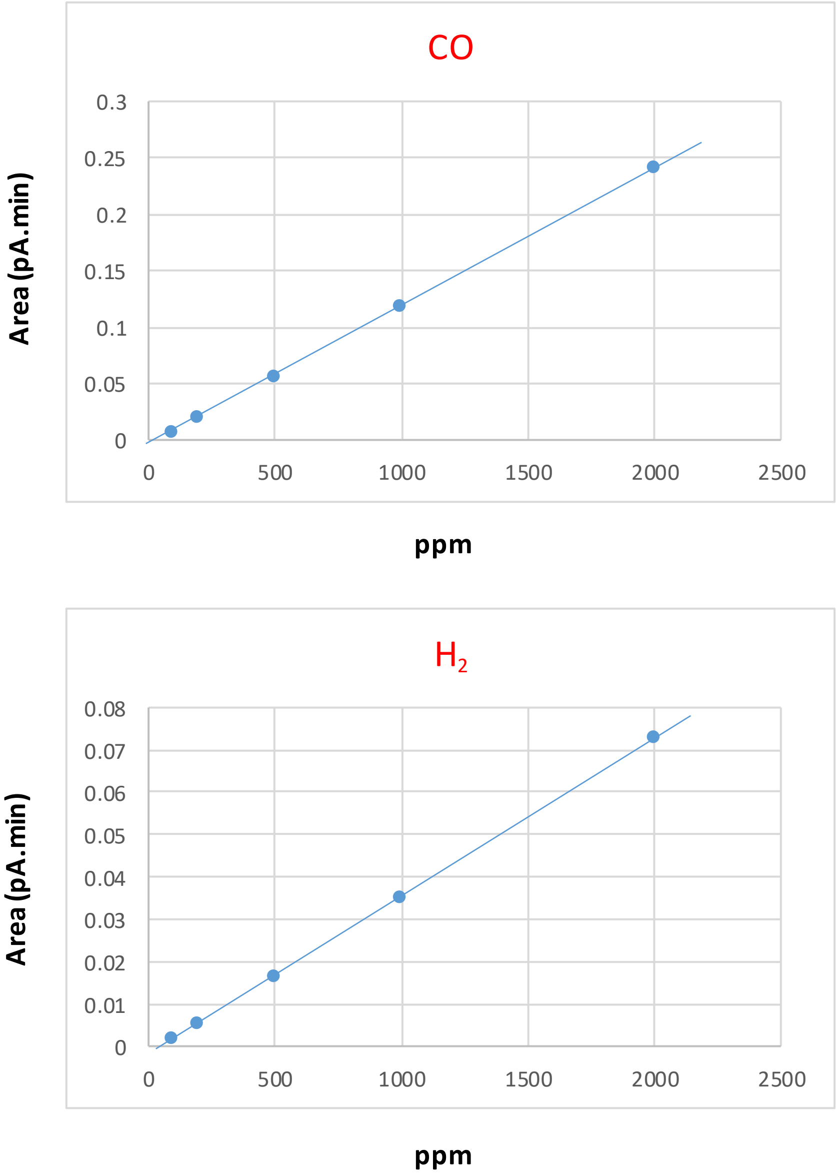

Figure S8. Calibration of the GC for H_2 and CO.

The pressure drop in GC was taken into account for calculating CO concentration. The *flow in* was always contrasted with the *flow out* to ensure a leakless experiment. Before electrolysis, a few sequences from the reactor were recorded to obtain the background with no reaction. After obtaining the background, electrolysis started and CO was detected (Figure S9). After GC measurement, the pressure drop was re-measured to make sure the same condition as the beginning of the electrolysis. Based on calibration measurements, CO ppm was evaluated and then converted to *mol* fraction by the use of GC pressure drop, lab temperature, and total volume of the gas. After electrolysis, the solution was analyzed by NMR to inspect the presence of other possible products.

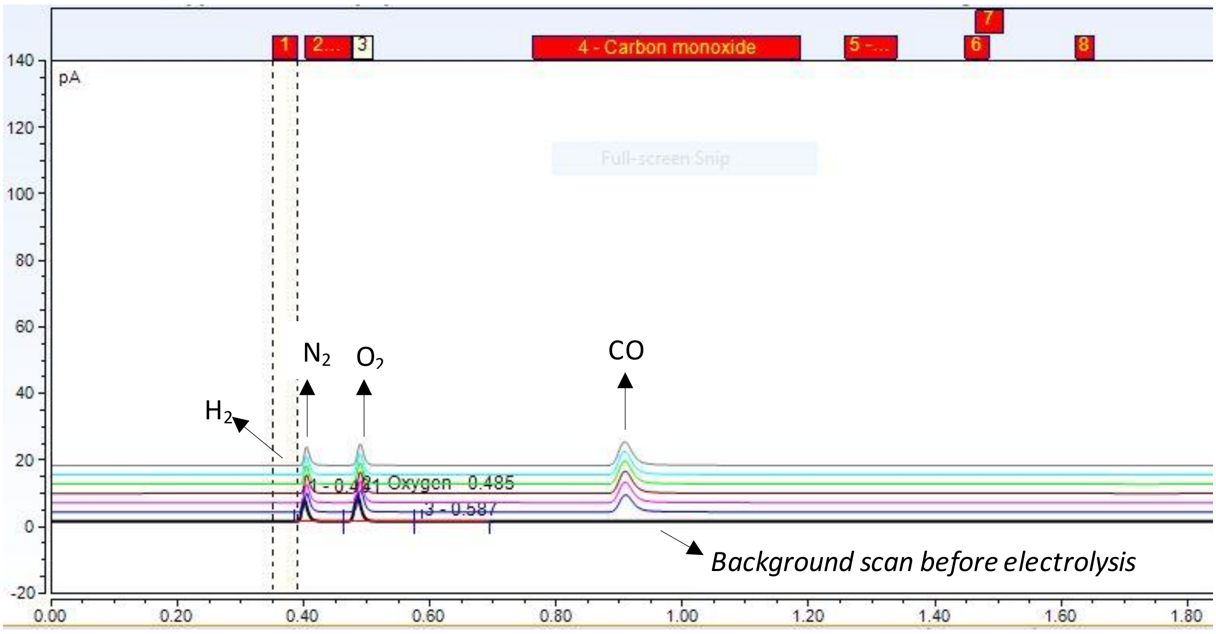

Figure S9. Subsequent sequences of the GC for detection of the products for CO_2 reduction. The black line is the background obtained before electrolysis. CO is the only product. The electrolyte was 0.5 mol% **MM** NTf_2 in anhydrous MeCN.

IV. VDD charge analysis of cations

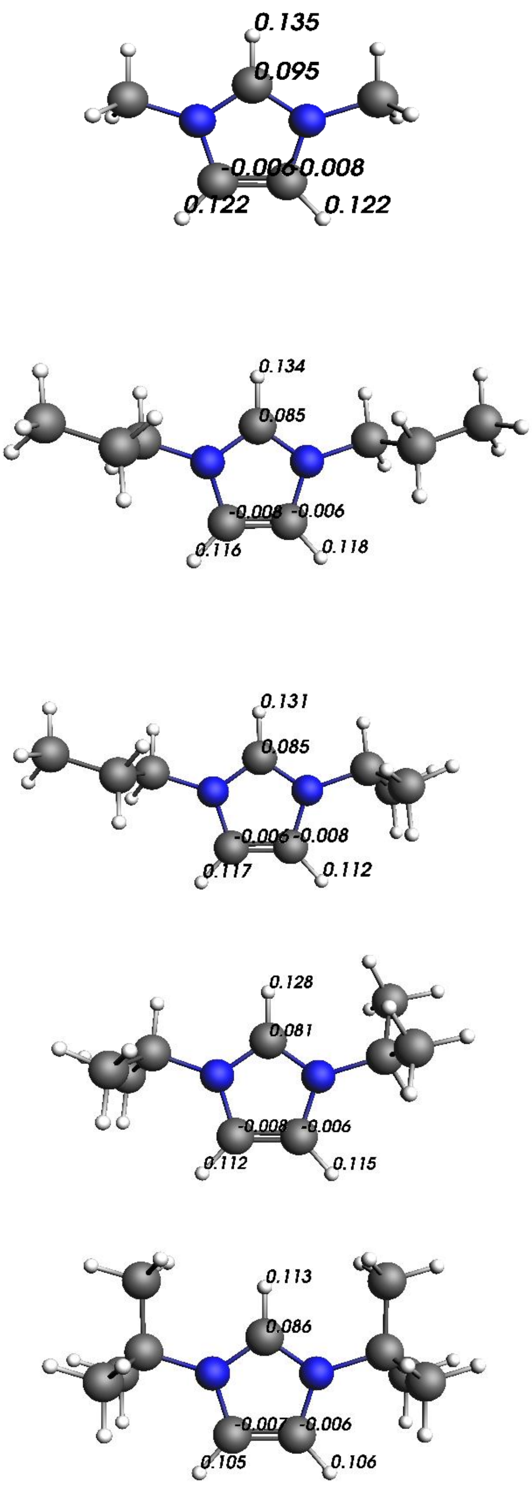

Figure S10. VDD charge analysis for imidazolium cations in acetonitrile.

V. ^{13}C NMR chemical shifts (ppm) of imidazolium NTf2 salts in CD3CN

Table S1. **^{13}C NMR chemical shifts (ppm) of imidazolium NTf_2 salts in CD_3CN and their activity for CO_2 reduction in acetonitrile.**

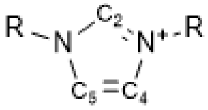

	C-2	C-4 + C-5	Current density at -2 V vs. Ag/Ag^{+}
Methyl Methyl	137.85	124.88	-1370
n-Propyl *n*-Propyl	136.59	123.85	-780
i-Propyl *n*-Propyl	135.33	121.93, 123.89	-385
i-Propyl *i*-Propyl	134.07	121.96	-245
t-Butyl *t*-Butyl	132.88	121.63	-65

VI. Stability of $^{*}CO_2^{-}$ vs the free CO_2 anion radical in acetonitrile

To evaluate the energy gain for CO_2 radical anion upon its binding with Au atom we calculated the formation energy for Au-$^{*}CO_2^{-}$ and $CO_2^{-\cdot}$ in acetonitrile and used the following equations:

$$G(*\,CO_2^{-})_{MeCN} = G(Au\_CO_2^{-})_{MeCN} - G(Au)_{MeCN}$$

$$\Delta G_{ads} = G(*\,CO_2^{-})_{MeCN} - G(CO_2^{\cdot-})_{MeCN} = 60.99 \text{ kcal/mol}$$

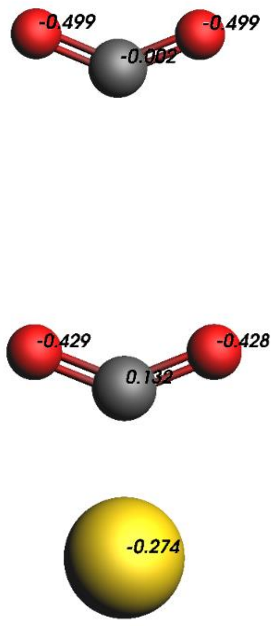

Figure S11. VDD charges for geometry optimized $*CO_2^{-}$ and $CO_2^{-\cdot}$ in acetonitrile. Upon binding Au atom a more uniform charge distribution is obtained. The formation energy of ~70 kcal/mol highlights the greater stability of $*CO_2^{-}$ versus $CO_2^{-\cdot}$.

VII. Interaction of C2-proton and C4, C5-protons of MM with Au- CO_2^-

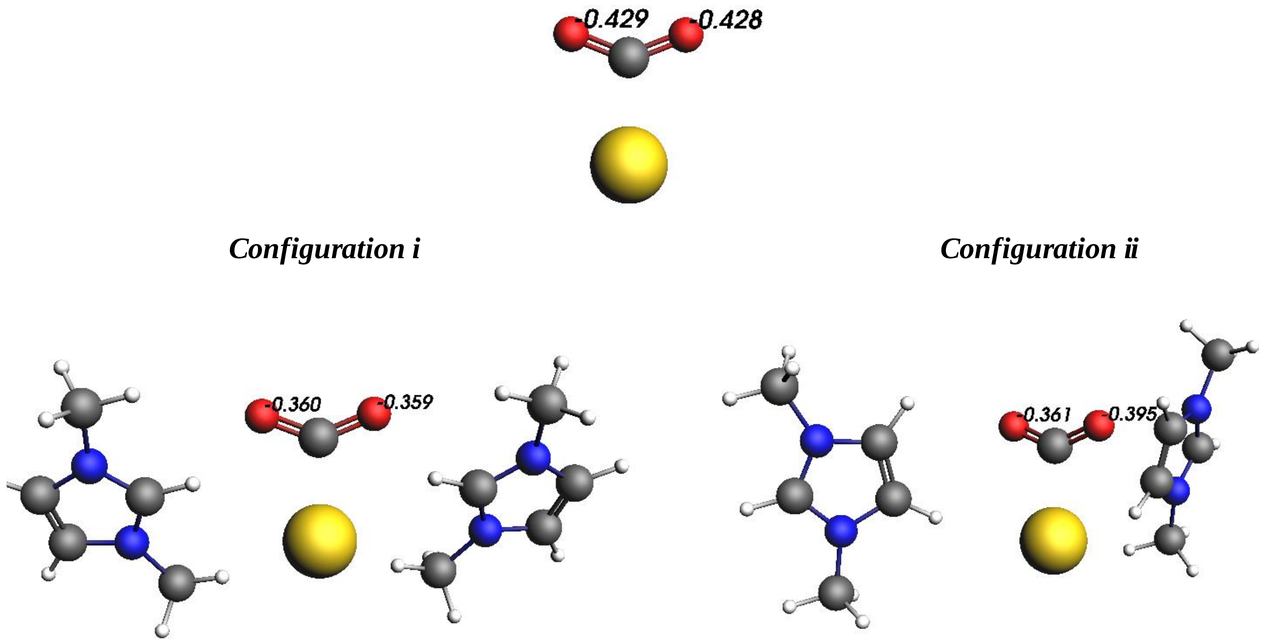

$Q(oxygen)_{Au-CO2} - Q(oxygen)_{Au-CO2\,MM} = -0.138$ $Q(oxygen)_{Au-CO2} - Q(oxygen)_{Au-CO2\,MM} = -0.101$

Figure S12. Differential VDD charge analysis for $^*CO_2^-$ in interaction with two possible configurations of the MM cation. Equations depict the charge difference between oxygen atoms in free Au-CO_2^- (top) and Au-CO_2^- in interaction with **MM** cations (bottom). Full VDD charges are provided in Figure S13.

Configuration i

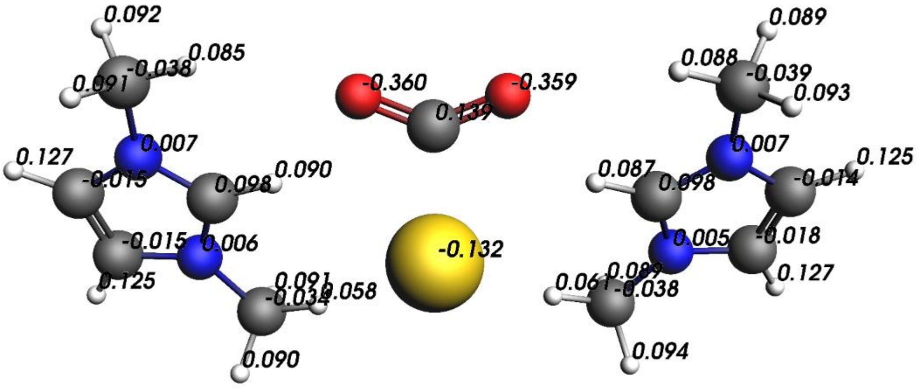

Configuration ii

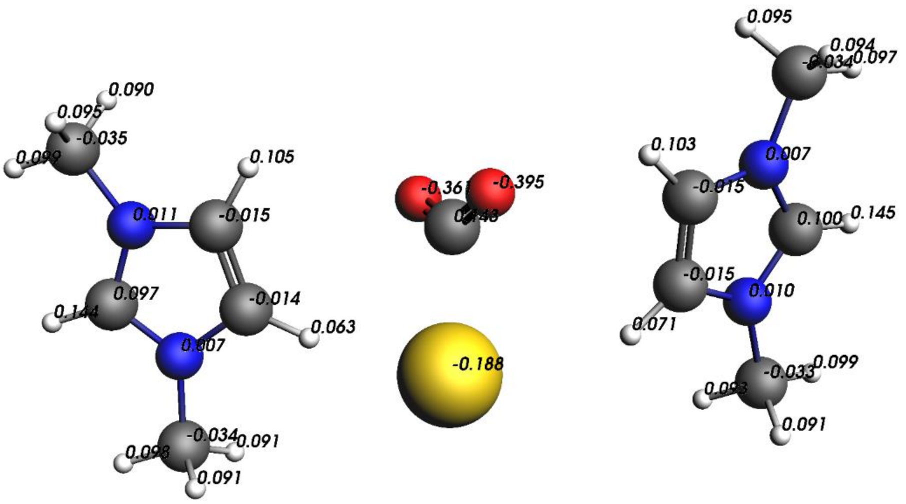

Figure S13. Charge distribution upon the interaction of $*CO_2^-$ and MM cation with two possible configurations. In *configuration i* C2-proton is pointed towards $*CO_2^-$ and in *configuration ii* C4- and C5-protons are positioned towards $*CO_2^-$. Full VDD charges are depicted.

VIII. Analysis of the activation energy for isomerization step

To investigate the proton transfer as the RDS in the isomerization step proposed by Wang et al.[1] (Scheme. S1), we performed the activation energy analysis for three cations: **MM**, **n-Pr n-Pr**, and **i-Pr i-Pr**.

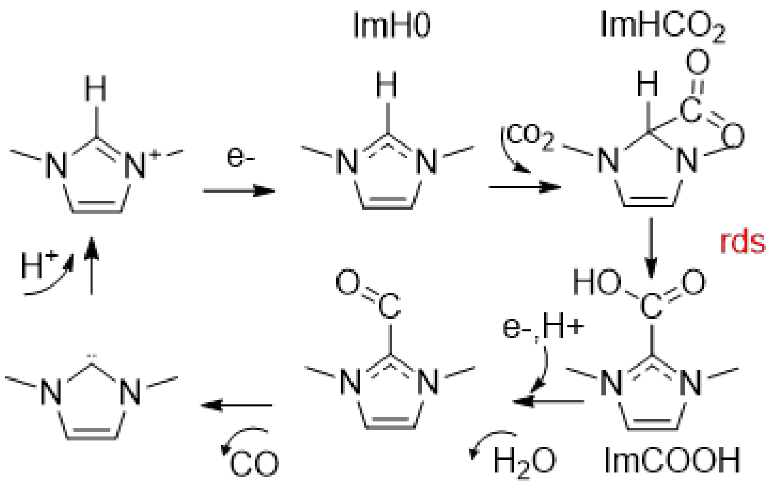

Scheme S1. Step-wise electron-proton transfer to CO2 mediated by imidazolium cation. Catalytic cycle proposed by Wang. et al.[1] for cation mediated sequential electron-proton transfer to CO_2. Proton transfer in the isomerization from $ImHCO_2$ adduct to ImCOOH was proposed as the rate-determining step.

The following steps were considered to evaluate the activation energy:

a) Geometries for cations (*compounds i* in Figure S14), reduced cations **ImH0** (*compounds ii* in Figure S14), **ImHCO$_2$** adducts (*compounds iii* in Figure S14), and intermediates **ImCOOH** (*compounds iv* in Figure S14) were optimized step-wise. Figure S14 shows the geometry optimized for all 4 compounds for **MM**, **n-Pr n-Pr**, and **i-Pr i-Pr** cations.

b) In order to find an initial guess for the transition state, we performed a potential energy surface scan (PES) for proton transfer proposed in the isomerization step (from *compound iii* to *compound iv*).

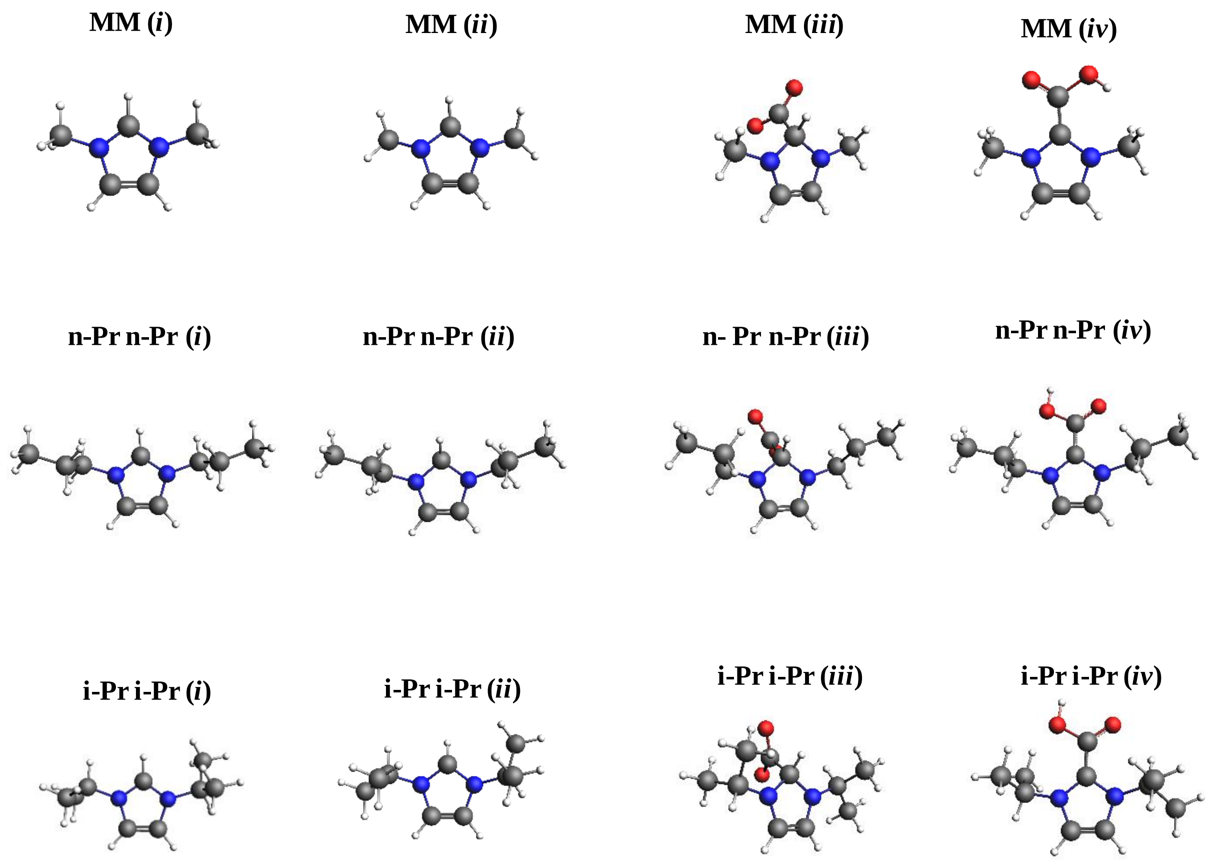

Figure S14. Optimized geometry for cations (*i*), reduced cations ImH0 (*ii*), ImHCO2 adduct (*iii*), and intermediates ImCOOH (*iv*) for three cations: MM, n-Pr n-Pr, i-Pr i-Pr. The energy was considered converged when the change in energy was smaller than 10^{-5} Hartree.

c) To make sure that the initial guess for the transition state from step **b** is positioned at a saddle point, we performed an extra transition state search with frequency calculations. The initial guess from step **b** was taken as the initial geometry for this calculation[2]. Geometries obtained for transition states are depicted in Figure S15. While PES was performed for multiple coordinates, for all cations the negative eigenvalue occurs for the vibration along the proton transfer.

MM TS

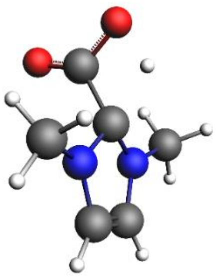

n-Pr n-Pr TS

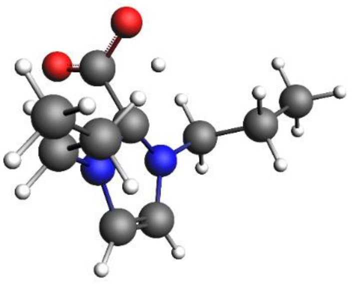

i-Pr i-Pr TS

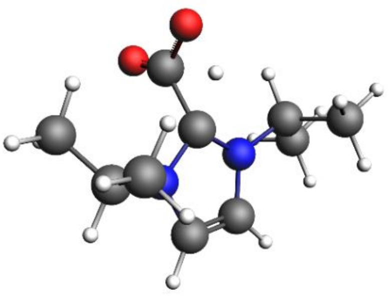

Figure S15. Transition state (TS) structures for the proton transfer in the isomerization step according to the electron-proton transfer mechanism proposed in scheme S1.

d) After finding the transition state, we obtained the reaction path with the intrinsic reaction coordinate (IRC) analysis[3-4]. With the IRC, the analysis starts at a transitions state and slides down the hill towards the adjacent local minimum at either side of the transition state. The IRC analysis gives full information about the pathway as well as the height of the barrier in the reaction[5]. For all three cations, the isomerization reaction is found to be endothermic and *compounds iii* and *compounds iv* are found as the stationary points for the reactants and products, respectively. Thus, the isomerization with proton transfer as the reaction coordinate remains the preferred pathway from *compound iii* to *compound iv* for all three cations. Table S2 reports the barrier height for all three cations. No noticeable difference is found for the three cations in terms of activation energy. This is in striking contrast with our electrochemical results and challenges the idea of isomerization as a rate-determining step.

Table S2. Activation energy for proton transfer in the isomerization step

Cation	ΔG^*
**MM**	46.11
**n-Pr n-Pr**	46.066
**i-Pr i-Pr**	46.098

IX. Notes on the initial act of the electrode

As argued in the article a first electron transfer to the imidazolium cation violates the role of the heterogeneous catalyst and the electrode turns into an electron supplier with some affinity for a given cation. That is similar to what has been proposed for the pyridinium chemistry at electrodes such as Pt[6-7]. For CO_2 reduction with pyridinium as a molecular electro-catalyst, the cation first reduces to form a radical active form, and with the follow-up electron-proton transfers, CO_2 is reduced to products such as methanol and formic acid. The CV profile for such a system shows the same onset potential for the reduction of the cation and the reduction of CO_2 (Figure S16 a). This implies that the first electron transfer to pyridinium is involved in the initial stages of the mechanism. However, with imidazolium, there is a significant difference between the onset potential for the reduction of the cation and the onset potential for the reduction of CO_2 (Figure S16 b). Even with seven times higher concentrations of MM compared to CO_2, we did not observe cation reduction in the potential region in which CO_2 reduction occurs. This observation challenges the idea of the first electron transfer to imidazolium as an initial step, otherwise, similar to pyridinium chemistry we would observe the same onset potentials for the reduction of the cation and the reduction of CO_2. Indeed, a proposed mechanism for imidazolium-assisted electrochemical CO_2 reduction should consider the role of the electrode catalyst in adsorbing CO_2 and facilitating electron transfer to adsorbed CO_2.

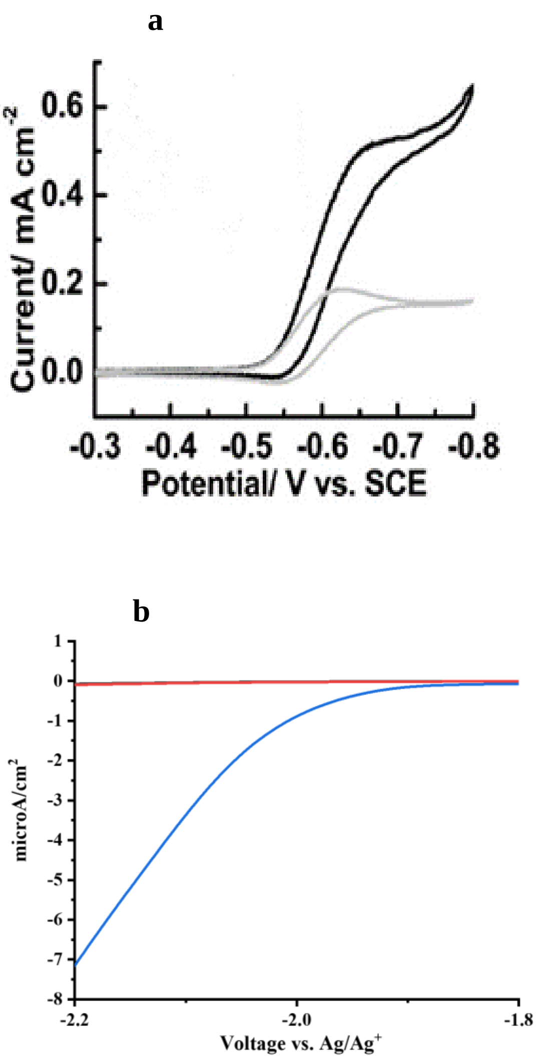

Figure S16. **a,** CVs of pyridinium reduction under Ar (**gray**) and CO_2 (**black**) at a Pt disk electrode in 0.5 M KCl aqueous electrolyte. **b,** LSVs for a Au disk electrode in acetonitrile with 0.5 mol% (**red**) and 1 mol% (**black**) of MM NTf_2 during He purging. (**blue**) is LSV with 0.5 mol% of MM under CO_2 purging. CO_2 solubility in our reactor condition is 0.14 mol%. Figure S16 **a** reprinted with permission from Ref S7. Copyright 2018, American Chemical Society.

X. Inverse kinetic isotope effect estimated from frequency calculations

To evaluate the kinetic isotope effect, we calculated IR frequencies for hydrogenated and deuterated MM cations. These frequencies were compared with those of Au-COOH and Au-COOD as intermediates. Differences in ZPEs were calculated as follows:

$$\frac{k_H}{k_D} = \frac{Ae^{-E_a(H)/RT}}{Ae^{-E_a(D)/RT}}$$

$$E_a(D) = \frac{1}{2}hc\ [\bar{v}(OD) - \bar{v}(C_2D)]$$
$$E_a(H) = \frac{1}{2}hc\ [\bar{v}(OH) - \bar{v}(C_2H)]$$

$$\frac{k_H}{k_D} = e^{\frac{hc}{2RT}[[(\bar{v}(OD)-\bar{v}(OH)]-[(\bar{v}(C_2D)-\bar{v}(C_2H)]]}$$

$$\frac{k_H}{k_D} = 0.735\ ,\ \ \frac{k_D}{k_H} = \ 1.36$$

XI. Extra notes on the inverse kinetic isotope effect

While for most coupled electron-proton transfer reactions a normal KIE effect (k_H/k_D>1)is expected[8-10], the inverse KIE (iKIE) observed in this work highlights a unique feature of the imidazolium-mediated anhydrous CO_2 reduction. The observed inverse kinetic isotope effect shares similarities with the proton transfer in catalytic reactions involving the formation of low-barrier hydrogen bonds (LBHBs). In catalytic reactions associated with LBHBs, a weak hydrogen bond in the ground state becomes a low barrier hydrogen bond (strong and short-distance hydrogen bond) in the transition state[11-13]. LBHBs are mostly observed in non-protic solvents. The energy released in forming the LBHB then contributes to lowering the activation barrier for the reaction[11]. In another word, this energy release can explain the preference for the CEPT pathway over the sequential pathway in anhydrous media (Figure 4). Developing the strong hydrogen bonds between negatively charged oxygen of $^*CO_2^{\delta-}$ and $^{\delta+}$H-C2 of MM in the transition state, thus, should have caused the inverse KIE observed in this work.

XII. Notes on the electrochemical cycle

To explain the formation of CO in anhydrous conditions, one needs to consider the presence of an oxygen acceptor. In anhydrous media, there are two possible oxygen acceptors: either dissolved CO_2, or the ppm level of residual water in anhydrous acetonitrile. In the case of CO_2 as the oxygen acceptor, CO_3^{2-} should form as a byproduct; as was also argued by Lau. et al[14], through the carbon dioxide radical anion. Alternatively, protons associated with residual water might act as the oxygen acceptor, producing additional water. We were not able to detect carbonate species in our small-scale reactor, and post-electrolysis NMR analysis shows no evidence of water formation (Figures. S33 and S34). Thus, to examine the above-mentioned possibilities a thorough study should be performed requiring very sensitive and dedicated analysis, which is planned for future work. Also, the anodic reaction occurring and closing the electrochemical cycle requires detailed further investigation.

XIII. Synthetic procedures

1-Isopropyl-3-propylimidazolium iodide. A 250 mL round bottom flask equipped with a magnetic stirring bar and reflux condenser was charged with 1-isopropylimidazole (5.185 g, 47.0 mmol) and $NaHCO_3$ (1.5 eq., 70.5 mmol, 5.92 g). Acetonitrile (100 mL) and 1-iodopropane (2 eq., 94 mmol, 16.0 g, 9.2 mL) were added and the mixture was stirred at 50 °C for 1 week under N_2. Acetonitrile was removed under vacuum and the residue was dissolved in CH_2Cl_2 (50 mL). The CH_2Cl_2 solution was separated from the formed salts which were subsequently washed with some CH_2Cl_2. Under stirring, diethyl ether (150 mL) was added to the combined CH_2Cl_2 solutions under stirring to precipitate the imidazolium iodide product. The flask was placed in a refrigerator overnight. The CH_2Cl_2/ether layer was removed and the liquid residue was washed with diethyl ether (50 mL) which was also removed. The residue was dried under a slow flow of N_2 and subsequently under vacuum (6 mbar, then 0.01 mbar), yielding 1-isopropyl-3-propylimidazolium iodide as a colorless liquid (12.77 g, 97%).

^{1}H NMR (CD_3CN, 400 MHz): δ (ppm) = 0.90 (t, CH_3, 3H), 1.52 (d, CH_3, 6H), 1.88 (m, CH_2, 2H), 4.17 (t, N-CH_2, 2H), 4.66 (m, CH, 1H), 7.51 (s, H-4 or H-5, 1H), 7.57 (s, H-4 or H-5, 1H), 9.23 (H-2, s, 1H). ^{13}C NMR (CD_3CN, 100 MHz): δ (ppm) = 11.31 (CH_2CH_3), 23.58 (CH_3), 24.51 (CH_2), 52.07 (N-CH_2), 54.19 (CH), 121.99 (C-4 or C-5), 123.75 (C-4 or C-5), 136.29 (C-2).

Counterion exchange. The following procedure was used to convert 1-isopropyl-3-propylimidazolium iodide, 1,3-diisopropylimidazolium chloride and 1,3-di-*tert*-butylimidazolium tetrafluoroborate into the corresponding NTf_2 salts. The imidazolium iodide, chloride, or tetrafluoroborate (18.65 mmol) was dissolved in CH_3CN (20 mL). To this solution, Li NTf_2 (20.52 mmol, 1.1 eq.) was added as a solid. Milli-Q water (20 mL) was added and the mixture was stirred overnight. CH_3CN was removed by a flow of N_2 flow. The resulting mixture was extracted three times with CH_2Cl_2. The combined organic layers were washed once with Milli-Q water to remove the last traces of lithium salts, dried on Na_2SO_4, evaporated

to dryness using a rotary evaporator, and further dried under vacuum (6 mbar, then 0.01 mbar). 1-Isopropyl-3-propylimidazolium NTf_2 and 1,3-diisopropylimidazolium NTf_2 were obtained as colorless liquids, 1,3-di-*tert*-butylimidazolium NTf_2 as a white solid, in yields of 99%.

1-Isopropyl-3-propylimidazolium bis(trifluoromethylsulfonyl)imide
^{1}H NMR (CD_3CN, 400 MHz): δ (ppm) = 0.92 (t, CH_3, 3H), 1.51 (d, CH_3, 6H), 1.87 (m, CH_2, 2H), 4.09 (t, *N*-CH_2, 2H), 4.59 (m, CH, 1H), 7.41 (s, H-4 or H-5, 1H), 7.48 (s, H-4 or H-5, 1H), 8.51 (H-2, s, 1H). ^{13}C NMR (CD_3CN, 100 MHz): δ (ppm) = 11.17 (CH_2CH_3), 23.18 (CH_3), 24.37 (CH_2), 52.52 (*N*-CH_2), 54.55 (CH), 116.57 (CF_3), 119.76 (CF_3), 121.93 (C-4 or C-5), 122.94 (CF_3), 123.89 (C-4 or C-5), 126.13 (CF_3), 135.33 (C-2). ^{19}F NMR (CD_3CN, 376 MHz): δ (ppm) = −80.09 (s, CF_3).

1,3-Diisopropylimidazolium bis(trifluoromethylsulfonyl)imide
^{1}H NMR (CD_3CN, 400 MHz): δ (ppm) = 1.51 (d, CH_3, 12H), 4.58 (m, CH, 2H), 7.47 (s, H-4+H-5, 2H), 8.53 (H-2, s, 1H). ^{13}C NMR (CD_3CN, 100 MHz): δ (ppm) = 23.18 (CH_3), 54.58 (CH), 116.57 (CF_3), 119.76 (CF_3), 121.96 (C-4+C-5), 122.94 (CF_3), 126.13 (CF_3), 134.07 (C-2). ^{19}F NMR (CD3CN, 376 MHz): δ (ppm) = −80.08 (s, CF_3).

1,3-Di-*tert*-butylimidazolium bis(trifluoromethylsulfonyl)imide
^{1}H NMR (CD_3CN, 400 MHz): δ (ppm) = 1.62 (s, $C(CH_3)_3$, 18H), 7.57 (s, H-4+H-5, 2H), 8.42 (H-2, s, 1H). ^{13}C NMR (CD_3CN, 100 MHz): δ (ppm) = 30.10 (CH_3), 61.56 (CH), 116.57 (CF_3), 119.75 (CF_3), 121.63 (C-4+C-5), 122.94 (CF_3), 126.13 (CF_3), 132.88 (C-2). ^{19}F NMR (CD_3CN, 376 MHz): δ (ppm) = −80.17 (s, CF_3).

1,3-Dimethyl-2,4,5-trideuteroimidazolium bis(trifluoromethylsulfonyl)imide. A mixture of 1,3-dimethylimidazolium bis(trifluoromethylsulfonyl)imide (5.0 g, 13.25 mmol), D_2O (6.0 mL, 0.332 mol) and triethylamine (0.1 mL, 0.72 mmol) was stirred at 65 °C for four days under N_2. After cooling, D_2O and Et_3N were removed under vacuum, fresh D_2O (6.0 mL) and triethylamine (0.1 mL, 0.72 mmol) were added, and stirring at 65 °C was continued for another four days. After a total of six heating-evaporation cycles, D_2O and Et_3N were removed under vacuum and the liquid residue was dissolved in CH_2Cl_2 and dried on Na_2SO_4. The dichloromethane was evaporated on a rotary evaporator, the residue was redissolved in 20 mL dichloromethane, passed through a regenerated cellulose membrane filter (pore size 0.2 µm) to remove traces of solids, and then dried under vacuum (6 mbar, then 0.01 mbar), yielding 1,3-dimethyl-2,4,5-trideuteroimidazolium bis(trifluoromethylsulfonyl)imide as a colorless liquid

(4.65 g, 92 %). ^{1}H NMR spectroscopy revealed a conversion of C2-H into C2-D of 97.7 % and of C4-H+C5-H into C4-D+C5-D of 94.1%.

^{1}H NMR (CD_3CN, 400 MHz): δ (ppm) = 3.81 (s, N-CH_3, 6H). Trace signals at δ = 7.31 (s, H-4+H-5) and δ = 8.35 (s, H-2) were visible in the spectrum. ^{13}C NMR (CD_3CN, 100 MHz): δ (ppm) = 37.01 (*N*-CH_3), 116.47 (CF_3), 119.66 (CF_3), 121.63 (C-4+C-5), 122.85 (CF_3), 124.17, 124.48, 124.67, 124.79 (C-4+C-5), 126.03 (CF_3), 137.23, 137.56, 137.89 (C-2). ^{19}F NMR (CD_3CN, 376 MHz): δ (ppm) = −80.18 (s, CF_3).

XIV. Copies of ^{1}H, ^{13}C and ^{19}F NMR spectra

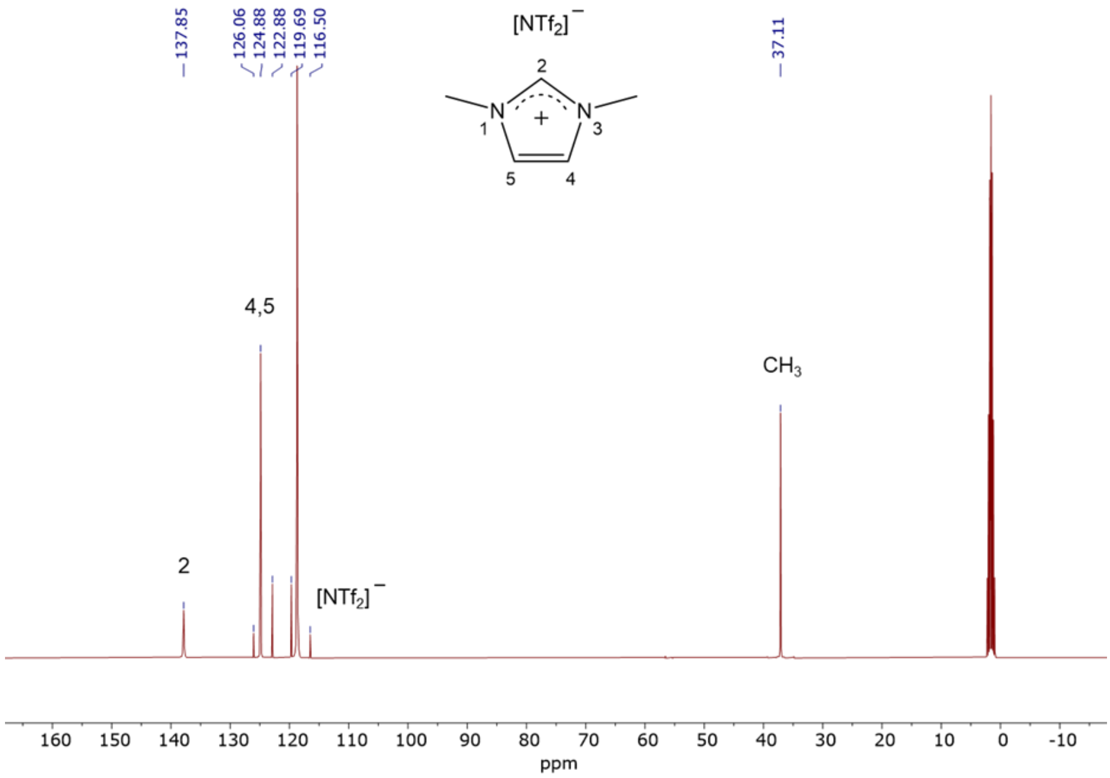

Figure S17. ^{13}C NMR spectrum of 1,3-dimethylimidazolium bis(trifluoromethylsulfonyl)imide in CD_3CN.

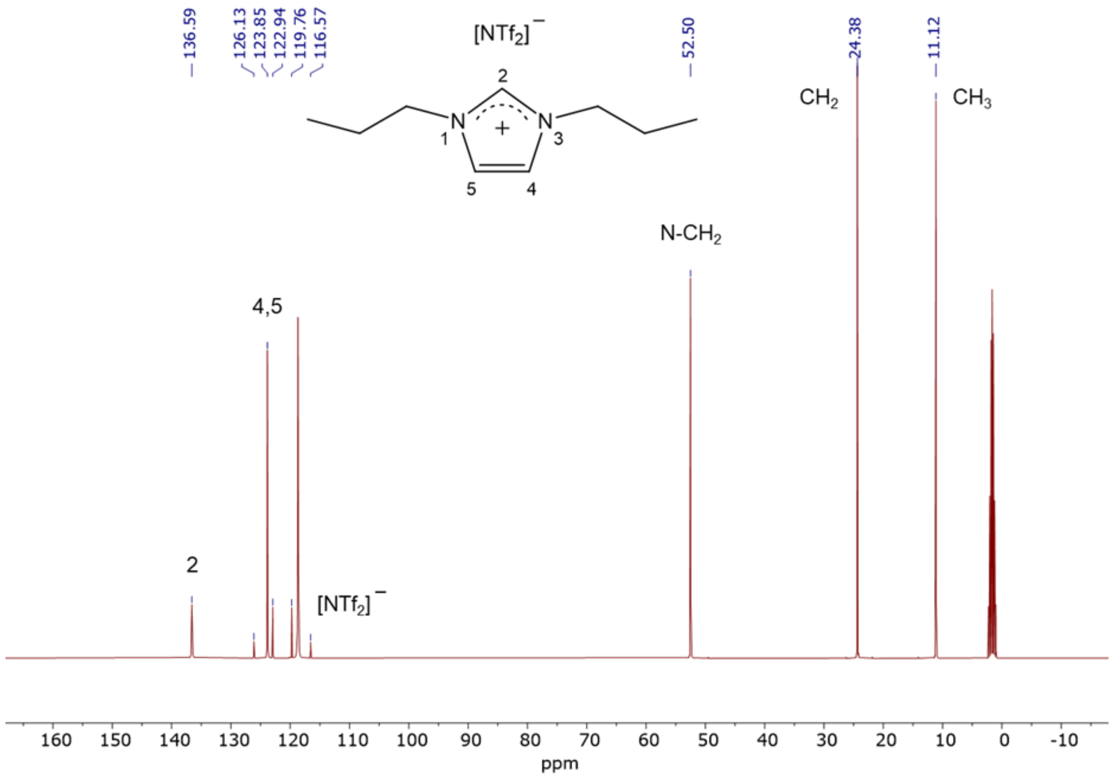

Figure S18. ^{13}C NMR spectrum of 1,3-dipropylimidazolium bis(trifluoromethylsulfonyl)imide in CD_3CN.

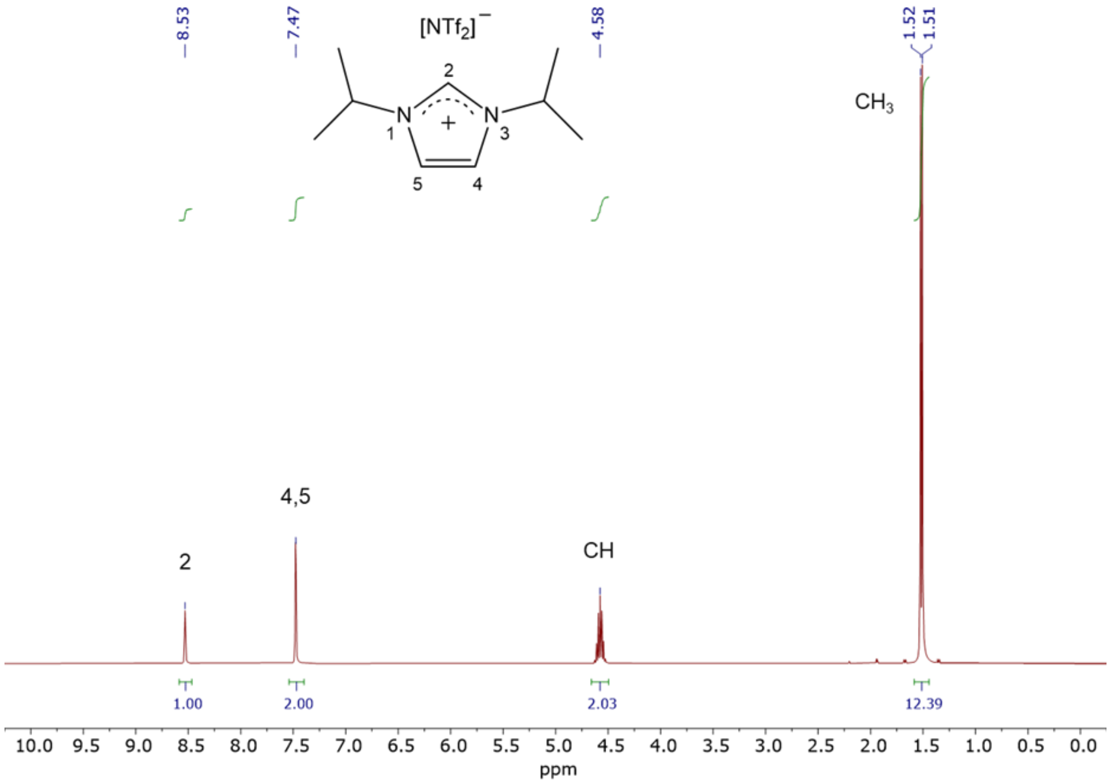

Figure S19. ^{1}H NMR spectrum of 1,3-diisopropylimidazolium bis(trifluoromethylsulfonyl)imide in CD_3CN.

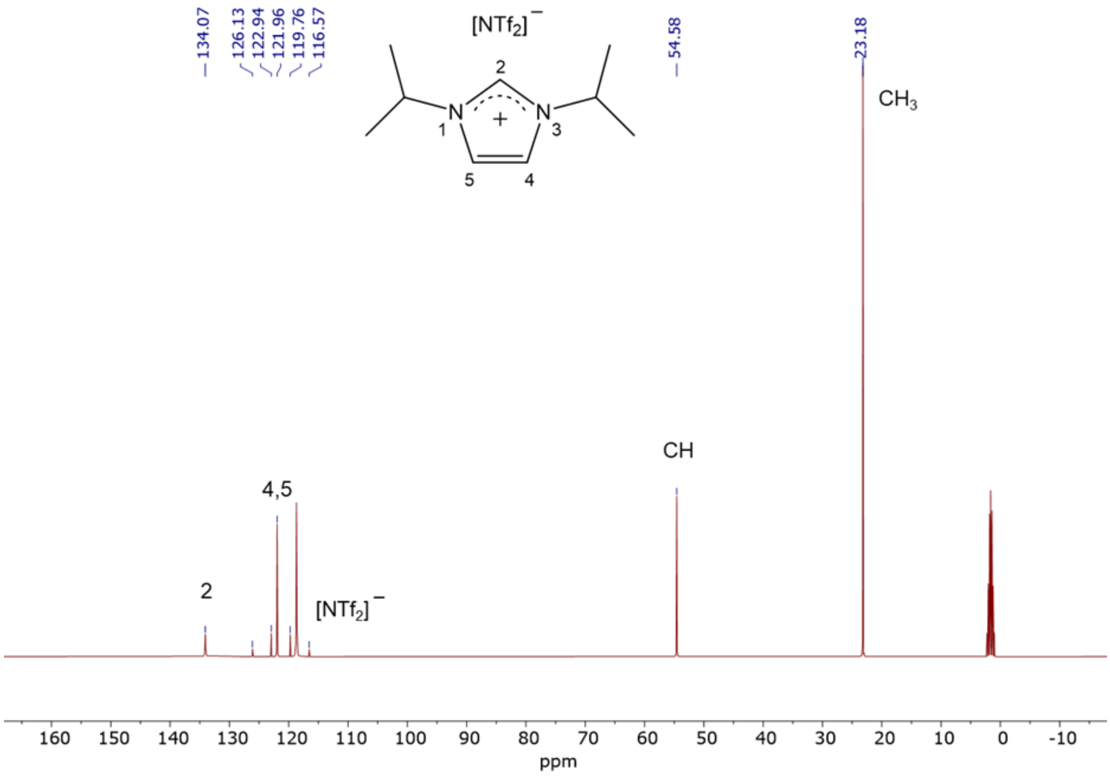

Figure S20. ^{13}C NMR spectrum of 1,3-diisopropylimidazolium bis(trifluoromethylsulfonyl)imide in CD_3CN.

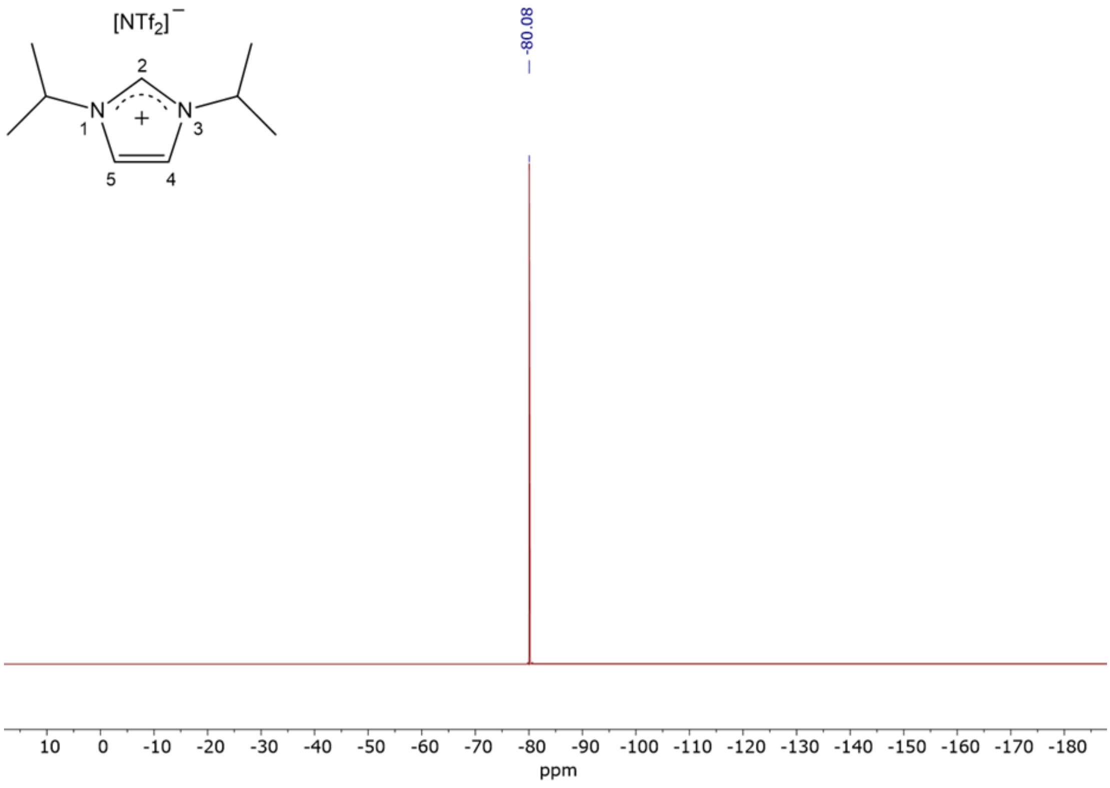

Figure S21. ^{19}F NMR spectrum of 1,3-diisopropylimidazolium bis(trifluoromethylsulfonyl)imide in CD_3CN.

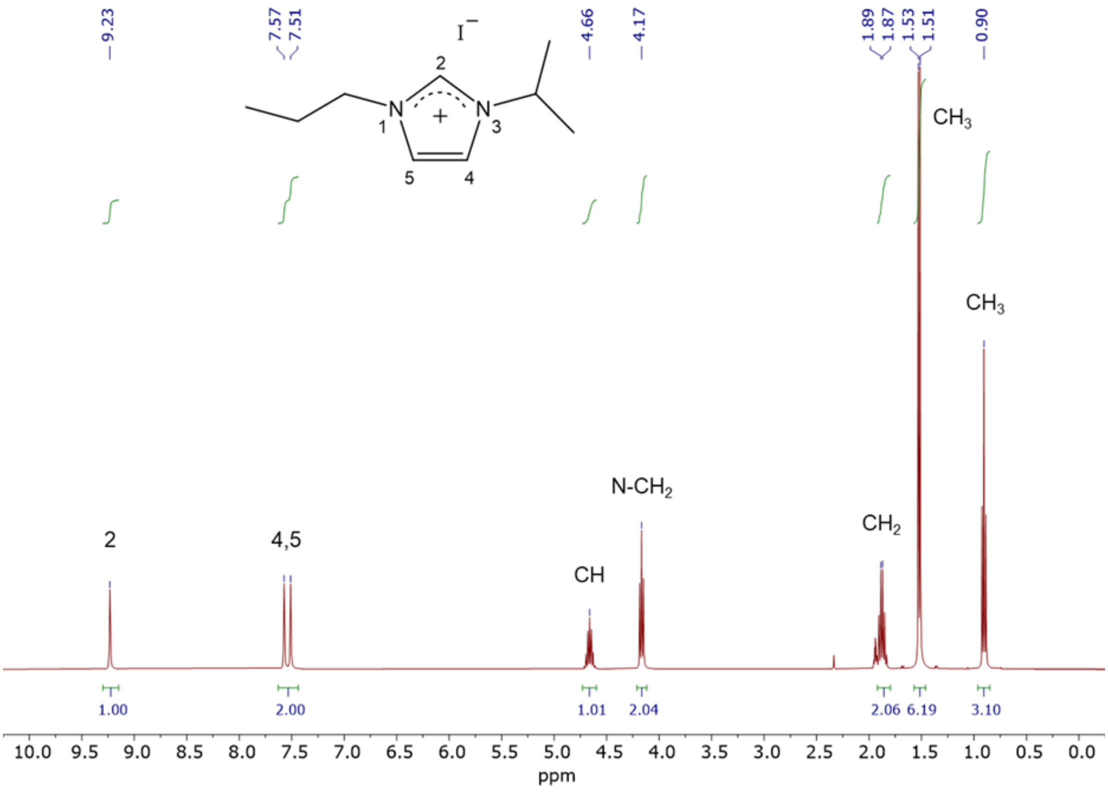

Figure S22. ^{1}H NMR spectrum of 1-propyl-3-isopropylimidazolium iodide in CD_3CN.

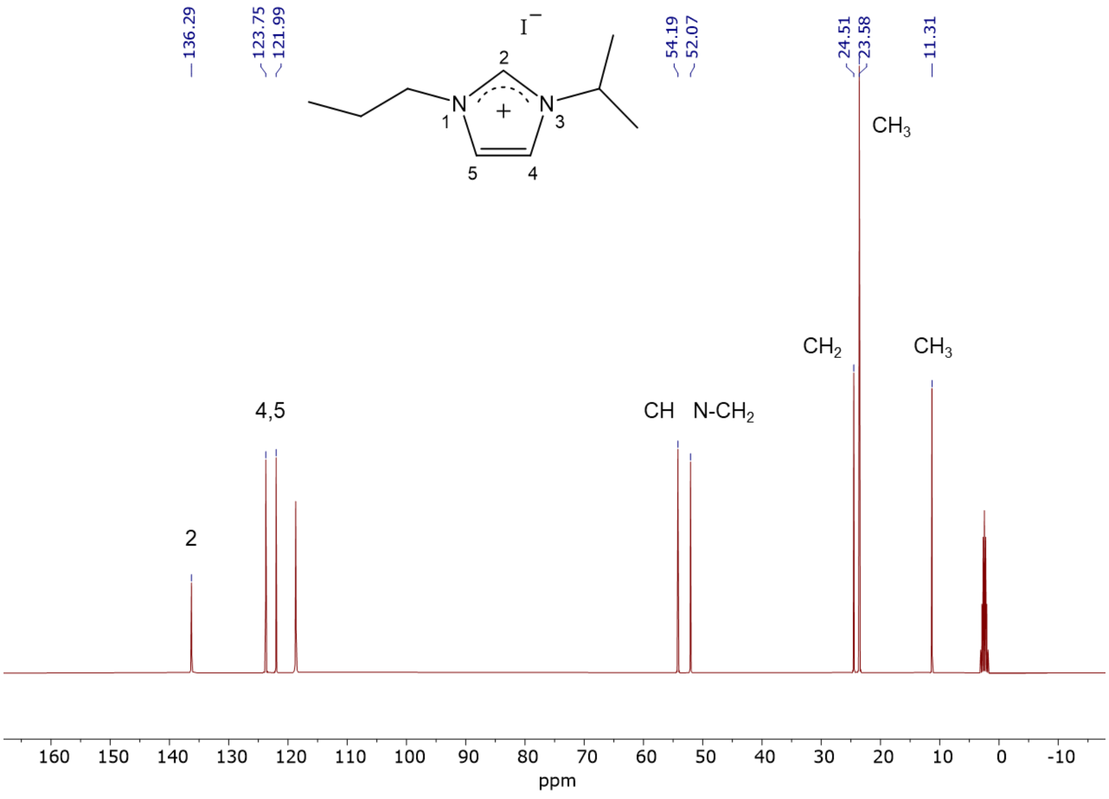

Figure S23. ^{13}C NMR spectrum of 1-propyl-3-isopropylimidazolium iodide in CD_3CN

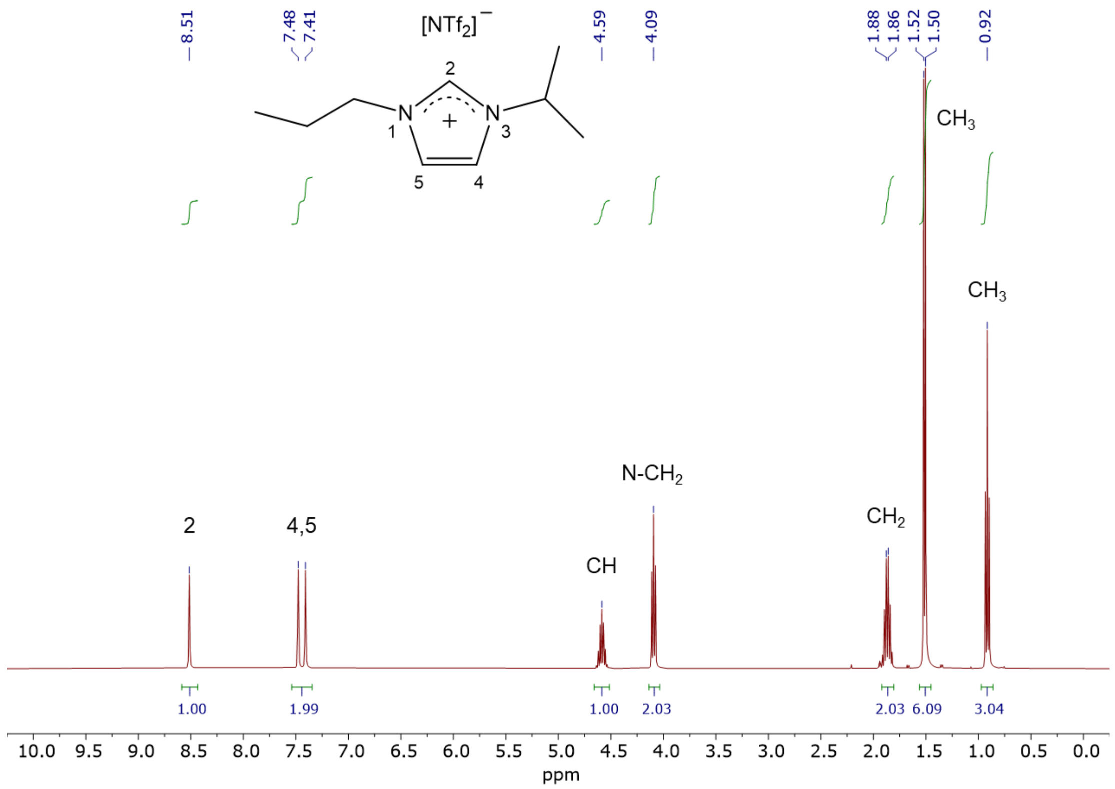

Figure S24. ¹H NMR spectrum of 1-propyl-3-isopropylimidazolium bis(trifluoromethylsulfonyl)imide in CD_3CN.

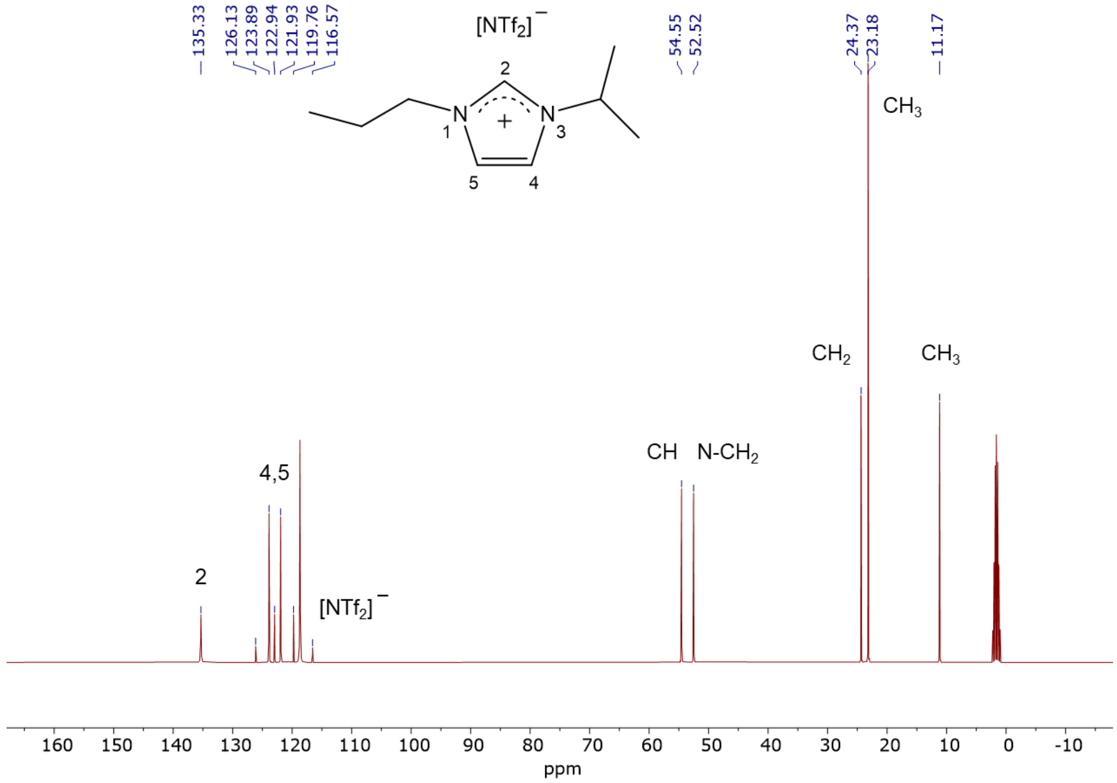

Figure S25. ¹³C NMR spectrum of 1-propyl-3-isopropylimidazolium bis(trifluoromethylsulfonyl)imide in CD_3CN.

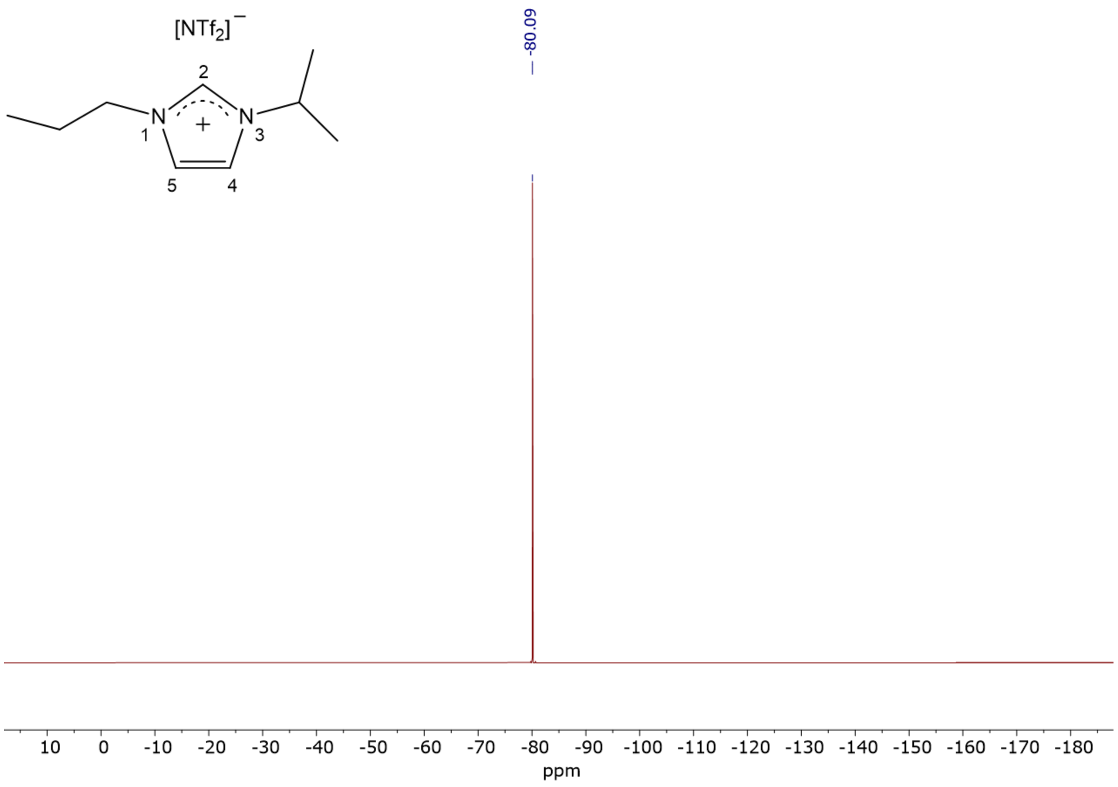

Figure S26. ^{19}F NMR spectrum of 1-propyl-3-isopropylimidazolium bis(trifluoromethylsulfonyl)imide in CD_3CN.

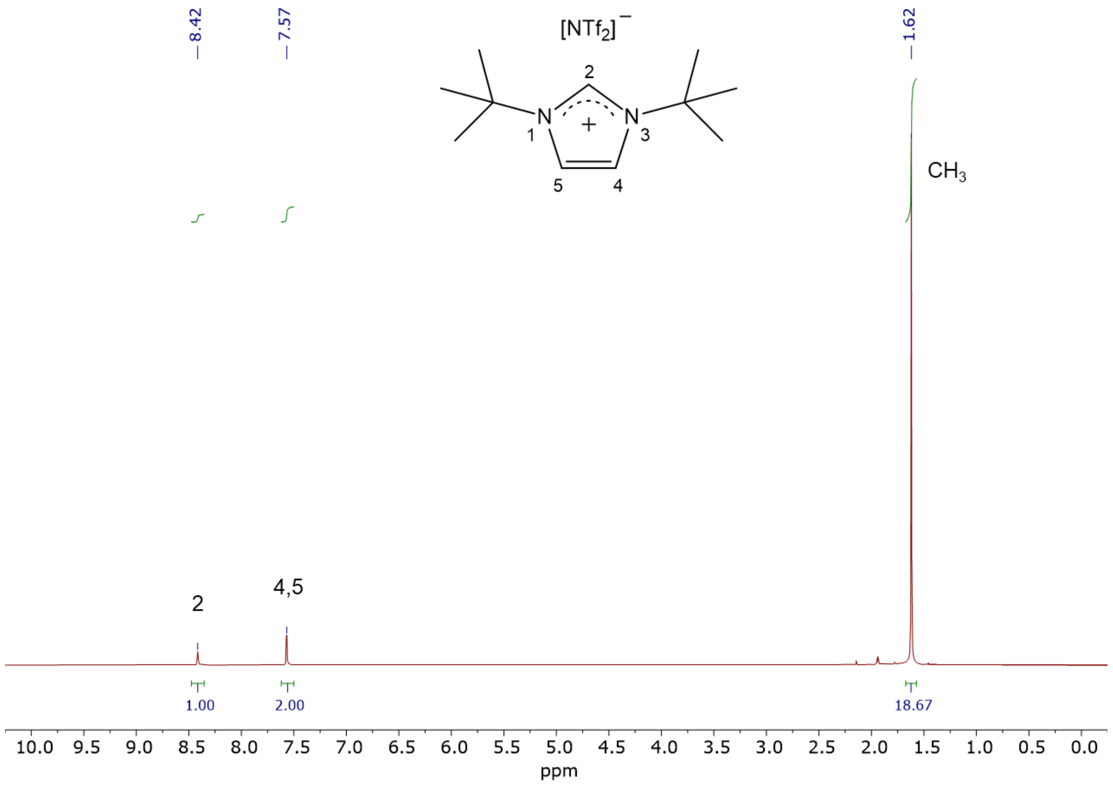

Figure S27. ^{1}H NMR spectrum of 1,3-di-*tert*-butylimidazolium bis(trifluoromethylsulfonyl)imide in CD_3CN.

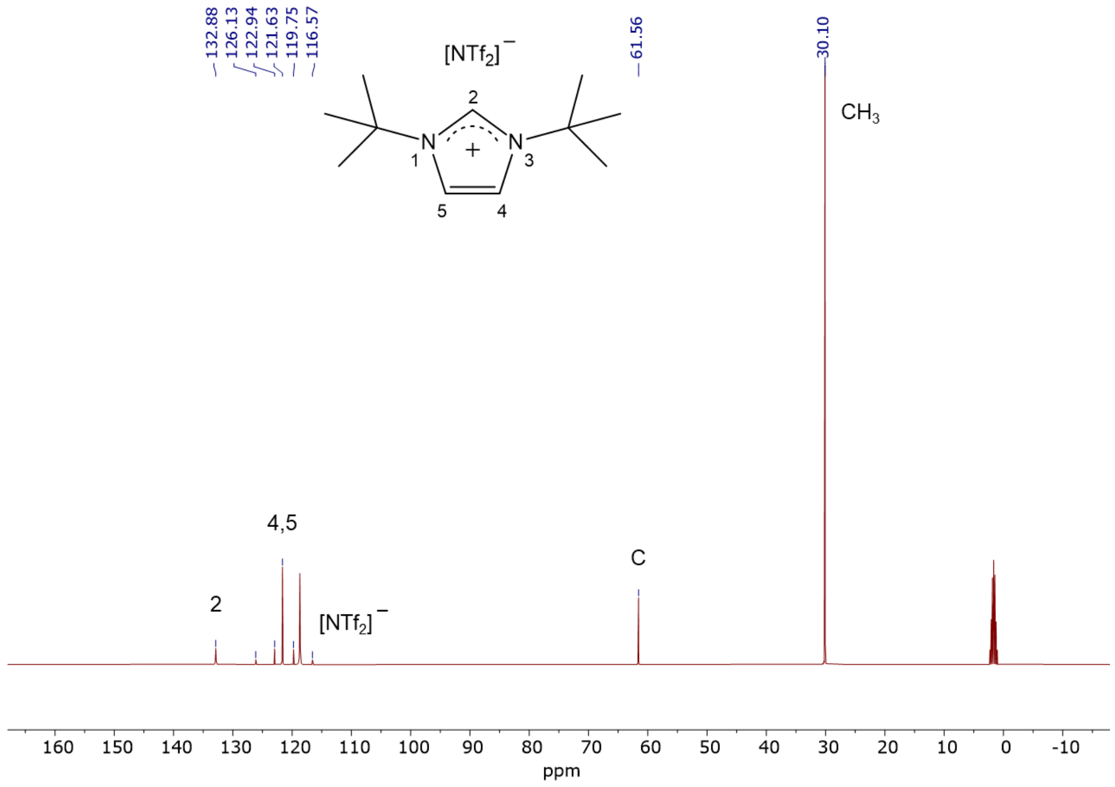

Figure S28. ^{13}C NMR spectrum of 1,3-di-*tert*-butylimidazolium bis(trifluoromethylsulfonyl)imide in CD_3CN.

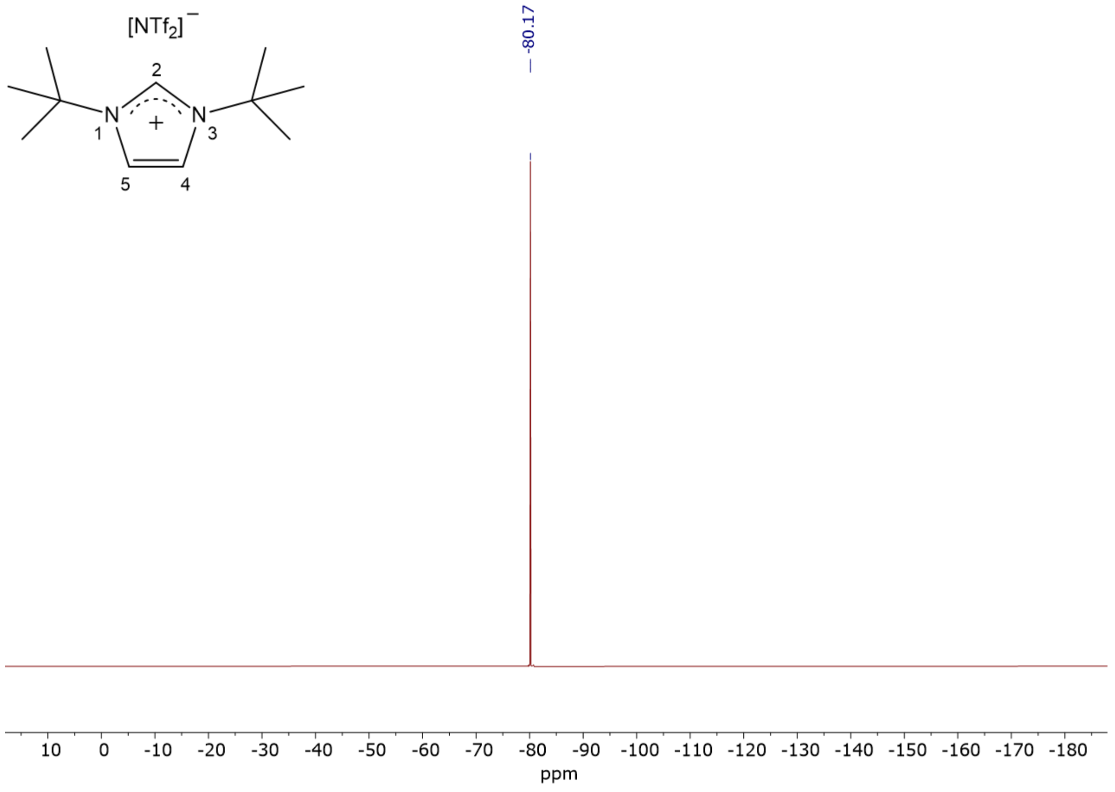

Figure S29. ^{19}F NMR spectrum of 1,3-di-*tert*-butylimidazolium bis(trifluoromethylsulfonyl)imide in CD_3CN.

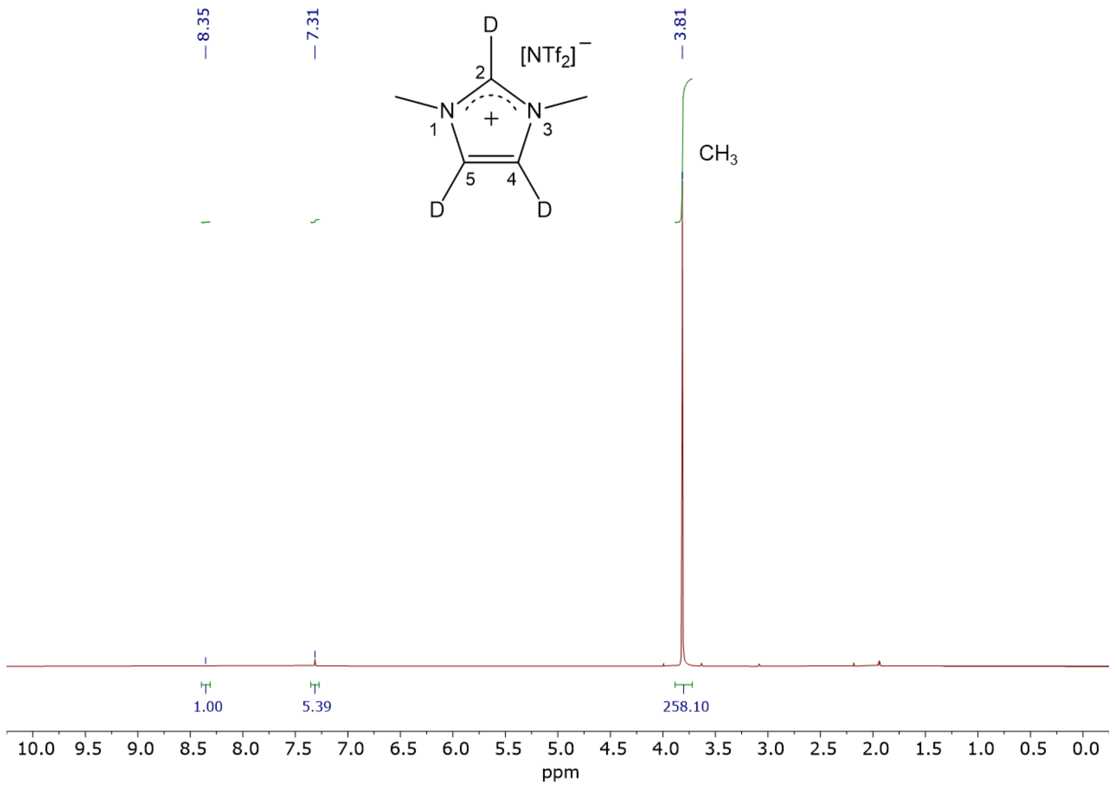

Figure S30. ^{1}H NMR spectrum of 1,3-dimethyl-2,4,5-trideuteroimidazolium bis(trifluoromethylsulfonyl)imide in CD_3CN.

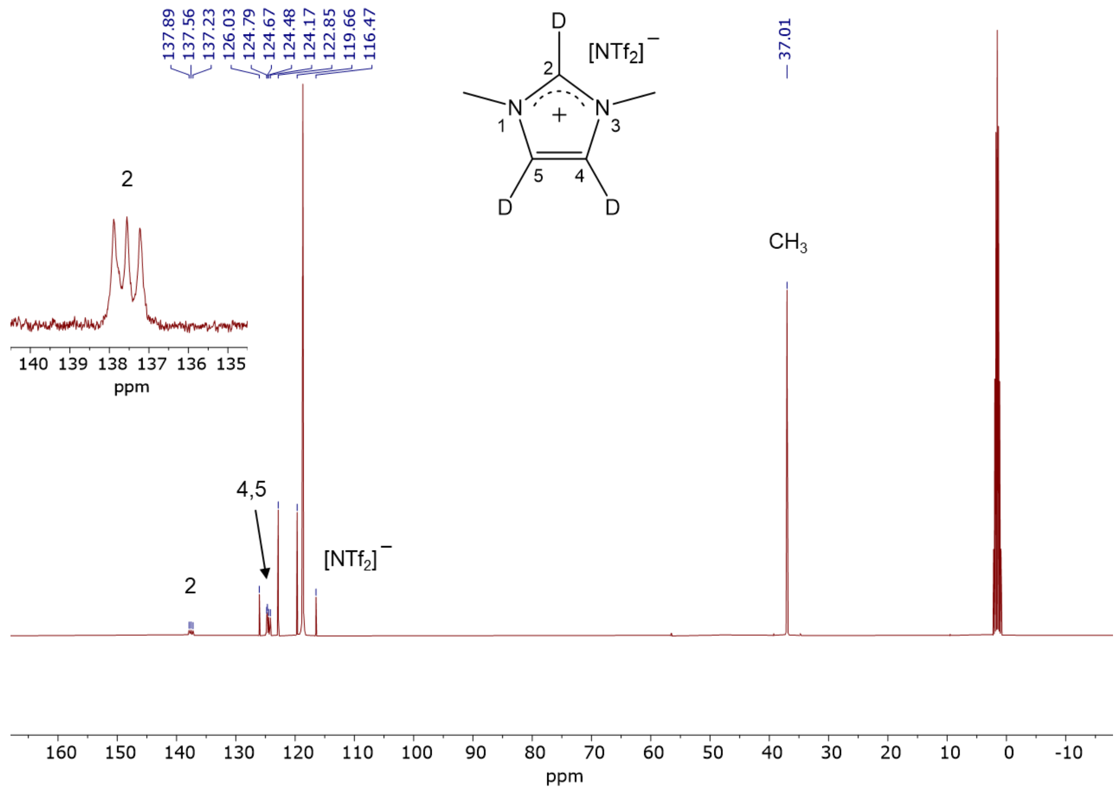

Figure S31. ^{13}C NMR spectrum of 1,3-dimethyl-2,4,5-trideuteroimidazolium bis(trifluoromethylsulfonyl)imide in CD_3CN.

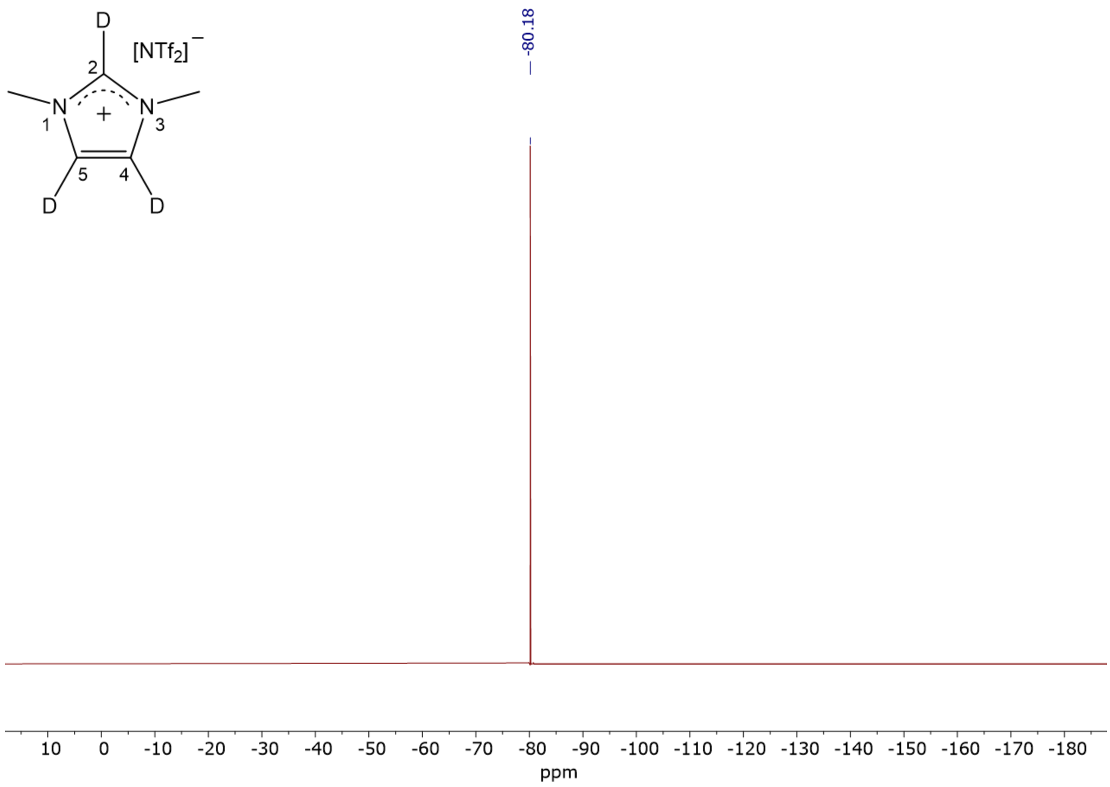

Figure S32. ^{19}F NMR spectrum of 1,3-dimethyl-2,4,5-trideuteroimidazolium bis(trifluoromethylsulfonyl)imide in CD_3CN.

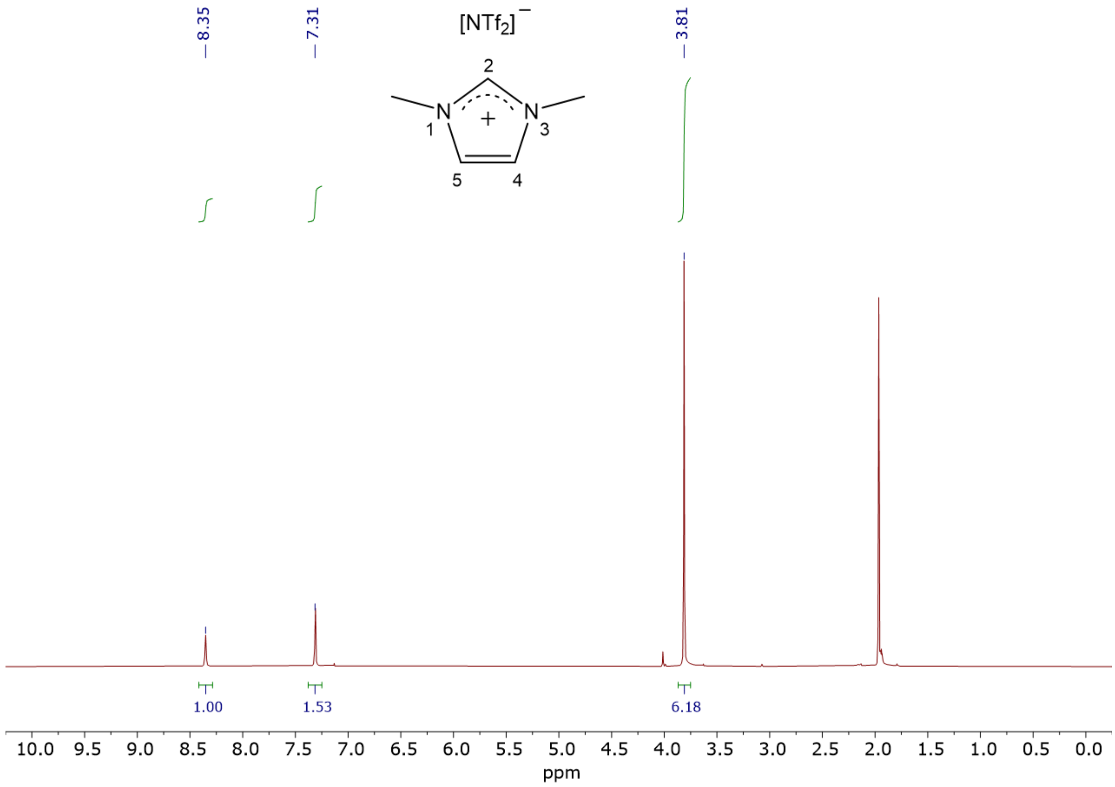

Figure S33. ^{1}H NMR spectrum of 1,3-dimethylimidazolium bis(trifluoromethylsulfonyl)imide in CD_3CN. The spectrum is from the MM-acetonitrile after 5 hrs. of electrolysis (Figure 1e).

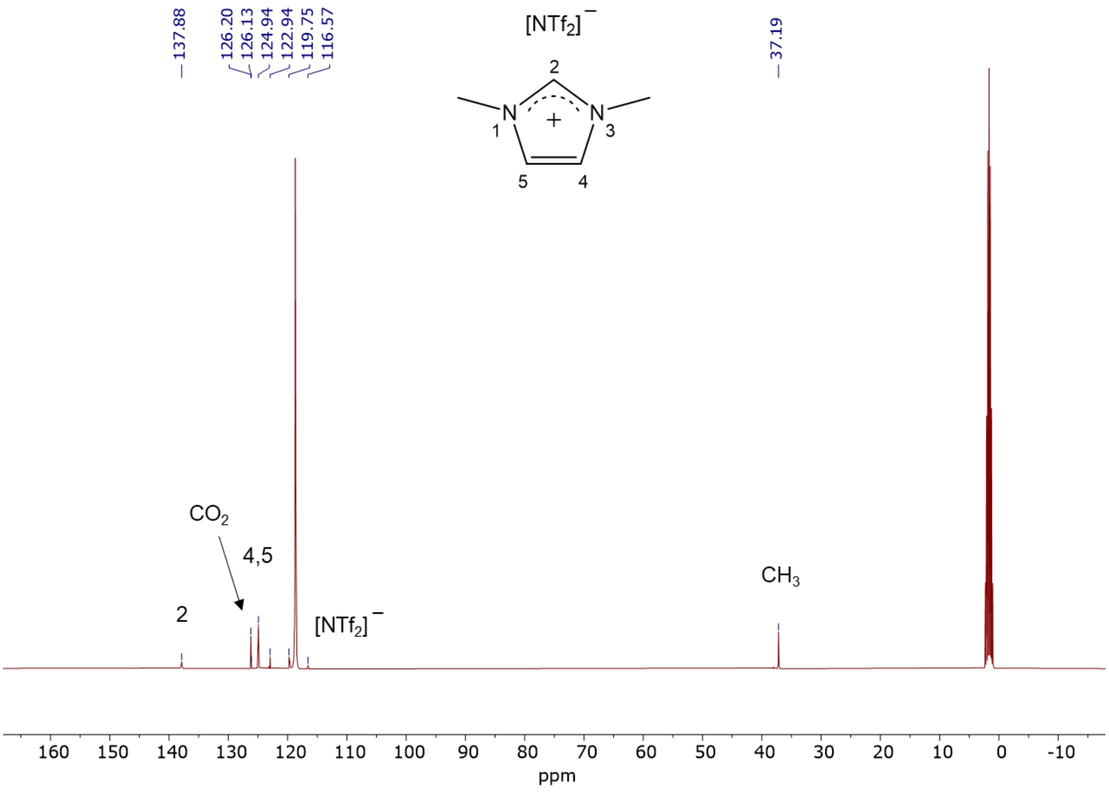

Figure S34. ^{13}C NMR spectrum of 1,3-dimethylimidazolium bis(trifluoromethylsulfonyl)imide in CD_3CN. The spectrum is from the MM-acetonitrile after 5 hrs. of electrolysis (Figure 1e). CO_2 peak appears as a result of CO_2 absorption by MM-acetonitrile.

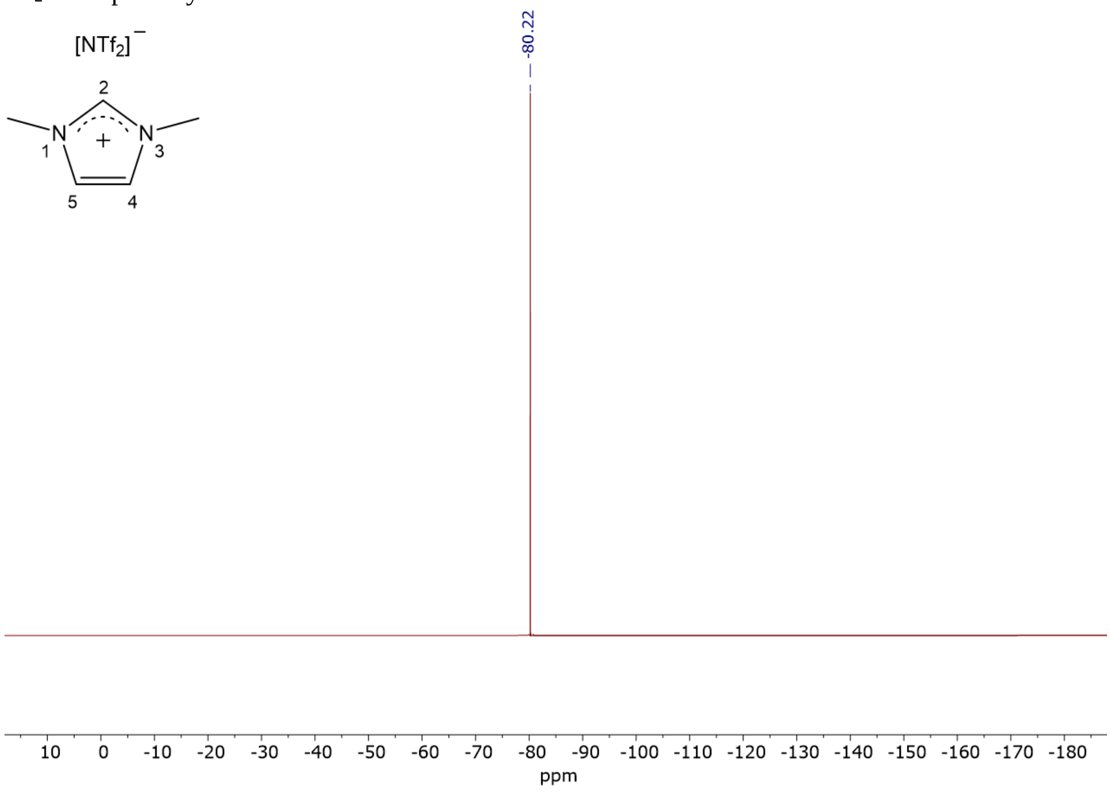

Figure S35. ^{19}F NMR spectrum of 1,3-dimethylimidazolium bis(trifluoromethylsulfonyl)imide in CD_3CN. The spectrum is from the MM-acetonitrile after 5 hrs. of electrolysis (Figure 1e).

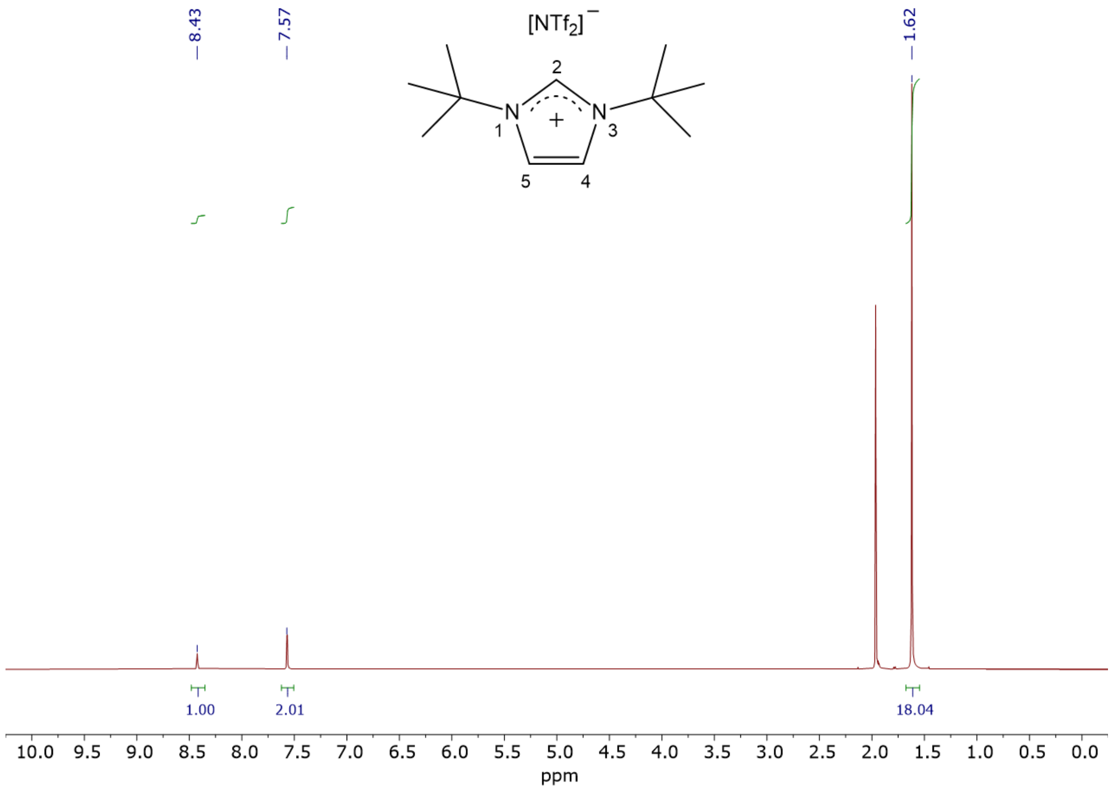

Figure S36. ^{1}H NMR spectrum of 1,3-di-*tert*-butylimidazolium bis(trifluoromethylsulfonyl)imide in CD_3CN The spectrum is from the t-Bu-acetonitrile after 1.5 hrs. of chronoamperometry at -1.8 V vs. Ag/Ag^+ (Figure S2).

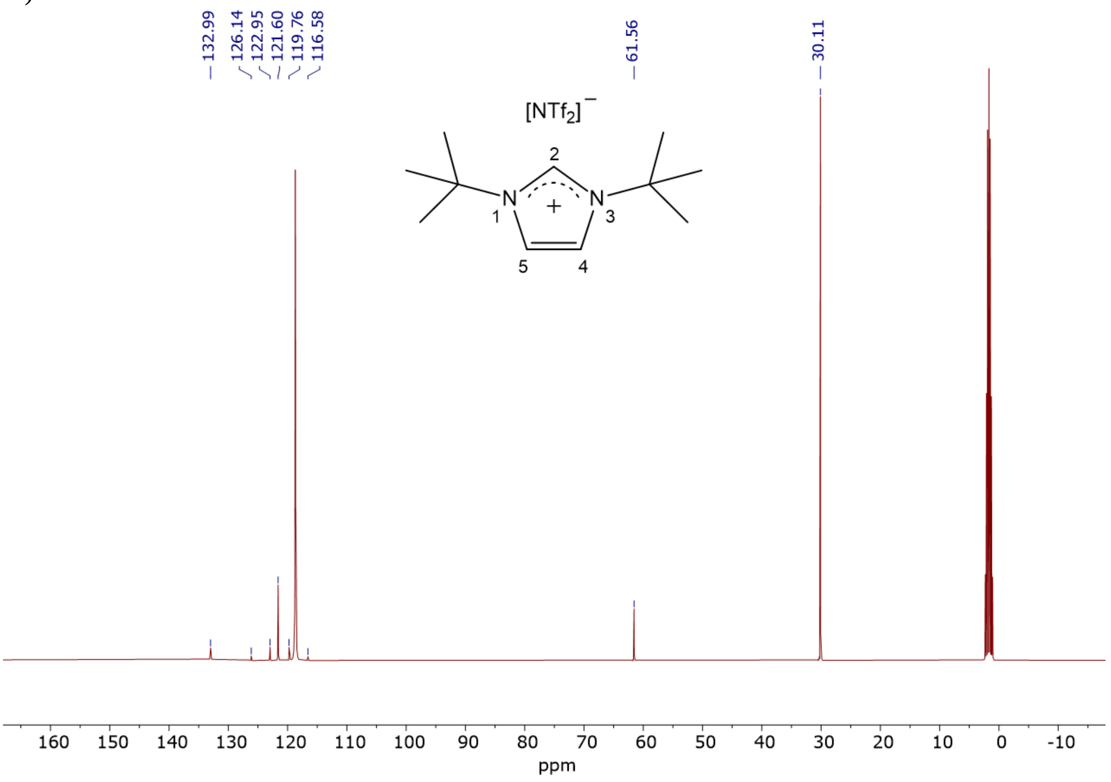

Figure S37. ^{13}C NMR spectrum of 1,3-di-*tert*-butylimidazolium bis(trifluoromethylsulfonyl)imide in CD_3CN The spectrum is from the t-Bu-acetonitrile after 1.5 hrs. of chronoamperometry at -1.8 V vs. Ag/Ag^+ (Figure S2).

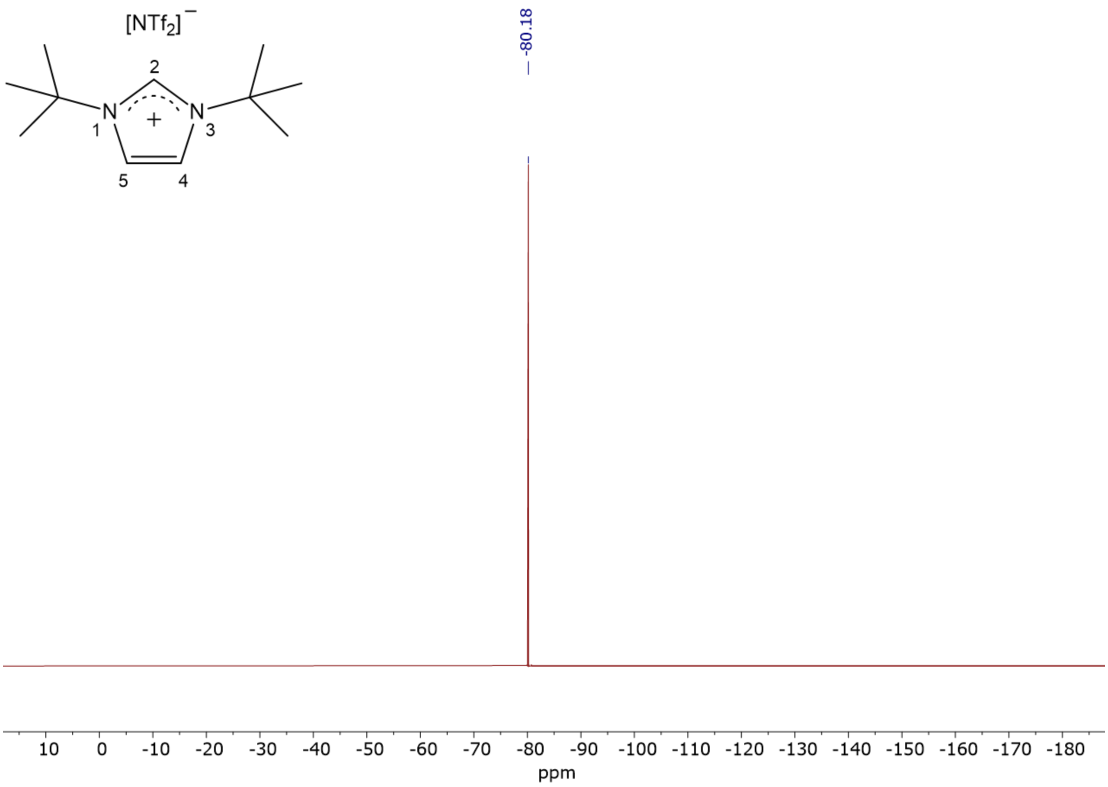

Figure S38. ^{19}F NMR spectrum of 1,3-di-*tert*-butylimidazolium bis(trifluoromethylsulfonyl)imide in CD_3CN The spectrum is from the t-Bu-acetonitrile after 1.5 hrs. of chronoamperometry at -1.8 V vs. Ag/Ag^+ (Figure S2).

XV. References

1. Wang, Y.; Hatakeyama, M.; Ogata, K.; Wakabayashi, M.; Jin, F.; Nakamura, S., Activation of CO2 by ionic liquid EMIM–BF4 in the electrochemical system: a theoretical study. *Physical Chemistry Chemical Physics* **2015,** *17* (36), 23521-23531.
2. Vermeeren, P.; van der Lubbe, S. C. C.; Fonseca Guerra, C.; Bickelhaupt, F. M.; Hamlin, T. A., Understanding chemical reactivity using the activation strain model. *Nature Protocols* **2020,** *15* (2), 649-667.
3. Deng, L.; Ziegler, T.; Fan, L., A combined density functional and intrinsic reaction coordinate study on the ground state energy surface of H2CO. *The Journal of Chemical Physics* **1993,** *99* (5), 3823-3835.
4. Deng, L.; Ziegler, T., The determination of intrinsic reaction coordinates by density functional theory. *International Journal of Quantum Chemistry* **1994,** *52* (4), 731-765.
5. te Velde, G.; Bickelhaupt, F. M.; Baerends, E. J.; Fonseca Guerra, C.; van Gisbergen, S. J. A.; Snijders, J. G.; Ziegler, T., Chemistry with ADF. *Journal of Computational Chemistry* **2001,** *22* (9), 931-967.
6. Barton Cole, E.; Lakkaraju, P. S.; Rampulla, D. M.; Morris, A. J.; Abelev, E.; Bocarsly, A. B., Using a One-Electron Shuttle for the Multielectron Reduction of CO2 to Methanol: Kinetic, Mechanistic, and Structural Insights. *Journal of the American Chemical Society* **2010,** *132* (33), 11539-11551.
7. Costentin, C.; Savéant, J.-M.; Tard, C., Catalysis of CO2 Electrochemical Reduction by Protonated Pyridine and Similar Molecules. Useful Lessons from a Methodological Misadventure. *ACS Energy Letters* **2018,** *3* (3), 695-703.
8. Reece, S. Y.; Nocera, D. G., Proton-coupled electron transfer in biology: results from synergistic studies in natural and model systems. *Annual review of biochemistry* **2009,** *78*, 673-99.
9. Mayer, J. M., PROTON-COUPLED ELECTRON TRANSFER: A Reaction Chemist's View. *Annual Review of Physical Chemistry* **2004,** *55* (1), 363-390.
10. Parada, G. A.; Goldsmith, Z. K.; Kolmar, S.; Rimgard, B. P.; Mercado, B. Q.; Hammarström, L.; Hammes-Schiffer, S.; Mayer, J. M., Concerted proton-electron transfer reactions in the Marcus inverted region. *Science* **2019,** *364* (6439), 471-475.
11. Cleland, W. W., Low-barrier hydrogen bonds and enzymatic catalysis. *Archives of biochemistry and biophysics* **2000,** *382* (1), 1-5.
12. Northrop, D. B., Follow the Protons: A Low-Barrier Hydrogen Bond Unifies the Mechanisms of the Aspartic Proteases. *Accounts of Chemical Research* **2001,** *34* (10), 790-797.
13. Fernandez, P. L.; Murkin, A. S., Inverse Solvent Isotope Effects in Enzyme-Catalyzed Reactions. *Molecules* **2020,** *25* (8), 1933.
14. Lau, G. P. S.; Schreier, M.; Vasilyev, D.; Scopelliti, R.; Grätzel, M.; Dyson, P. J., New Insights Into the Role of Imidazolium-Based Promoters for the Electroreduction of CO2 on a Silver Electrode. *Journal of the American Chemical Society* **2016,** *138* (25), 7820-7823.
